# Genomics-driven discovery of chiral triscatechol siderophores with enantiomeric Fe(iii) coordination†

**DOI:** 10.1039/d1sc03541j

**Published:** 2021-08-25

**Authors:** Parker R. Stow, Zachary L. Reitz, Timothy C. Johnstone, Alison Butler

**Affiliations:** Department of Chemistry and Biochemistry, University of California Santa Barbara CA 93106-9510 USA butler@chem.ucsb.edu; Department of Chemistry and Biochemistry, University of California Santa Cruz CA 95064 USA

## Abstract

Ferric complexes of triscatechol siderophores may assume one of two enantiomeric configurations at the iron site. Chirality is known to be important in the iron uptake process, however an understanding of the molecular features directing stereospecific coordination remains ambiguous. Synthesis of the full suite of (DHB^L/D^Lys^L/D^Ser)_3_ macrolactone diastereomers, which includes the siderophore cyclic trichrysobactin (CTC), enables the effects that the chirality of Lys and Ser residues exert on the configuration of the Fe(iii) complex to be defined. Computationally optimized geometries indicate that the Λ/Δ configurational preferences are set by steric interactions between the Lys sidechains and the peptide backbone. The ability of each (DHB^L/D^Lys^L/D^Ser)_3_ diastereomer to form a stable Fe(iii) complex prompted a genomic search for biosynthetic gene clusters (BGCs) encoding the synthesis of these diastereomers in microbes. The genome of the plant pathogen *Dickeya chrysanthemi* EC16 was sequenced and the genes responsible for the biosynthesis of CTC were identified. A related but distinct BGC was identified in the genome of the opportunistic pathogen *Yersinia frederiksenii* ATCC 33641; isolation of the siderophore from *Y. frederiksenii* ATCC 33641, named frederiksenibactin (FSB), revealed the triscatechol oligoester, *linear*-(DHB^L^Lys^L^Ser)_3_. Circular dichroism (CD) spectroscopy establishes that Fe(iii)–CTC and Fe(iii)–FSB are formed in opposite enantiomeric configuration, consistent with the results of the ferric complexes of the cyclic (DHB^L/D^Lys^L/D^Ser)_3_ diastereomers.

## Abstract 2

Synthesis and genome mining leverage access to diastereomers of the siderophore cyclic trichrysobactin. Computational modeling and CD spectroscopy address effects that ligand-based stereochemistry exerts on the configuration at Fe(iii).

## Graphical abstract

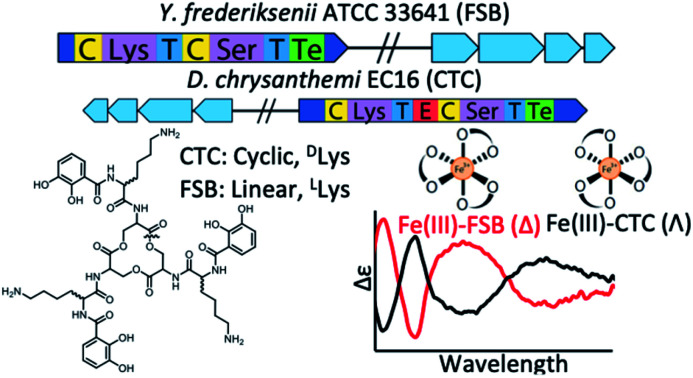

## Introduction

Chirality is universally significant in biological reactions, including the essential microbial process of iron acquisition mediated by small-molecule chelators known as siderophores. The specific three-dimensional structure of an Fe(iii)–siderophore complex plays a role in the ability of a bacterium to recognize, acquire, and extract iron from it.^1,2^ The triscatechol siderophores enterobactin (Ent) and bacillibactin (BB) each coordinate Fe(iii) with three 2,3-dihydroxybenzoyl (DHB) ligands framed on a macrolactone derived from three ^L^Ser or ^L^Thr residues, respectively (ESI Fig. S1†). Unlike Ent, BB also contains a glycine residue inserted between the macrolactone core and DHB. In stark contrast to Fe(iii)–Ent^3−^, which forms exclusively in the Δ configuration,^3,4^ Fe(iii)–BB^3−^ adopts the opposing Λ configuration.^3^ Several related triscatechol siderophores are further distinguished from Ent and BB by the presence of a chiral amino acid inserted between DHB and the oligoester backbone, including cyclic trichrysobactin [CTC; *Dickeya chrysanthemi* EC16] with ^D^Lys,^5^ as well as the linear tris-^L^Ser scaffolds of trivanchrobactin with ^D^Arg,^6^ and turnerbactin with ^L^Orn.^7^ Structurally, the influence amino acids exert on the configuration at the Fe(iii) site is incompletely understood; evolutionarily, these structural differences hint that chirality confers a competitive advantage in microbial iron uptake.

**Fig. 1:**
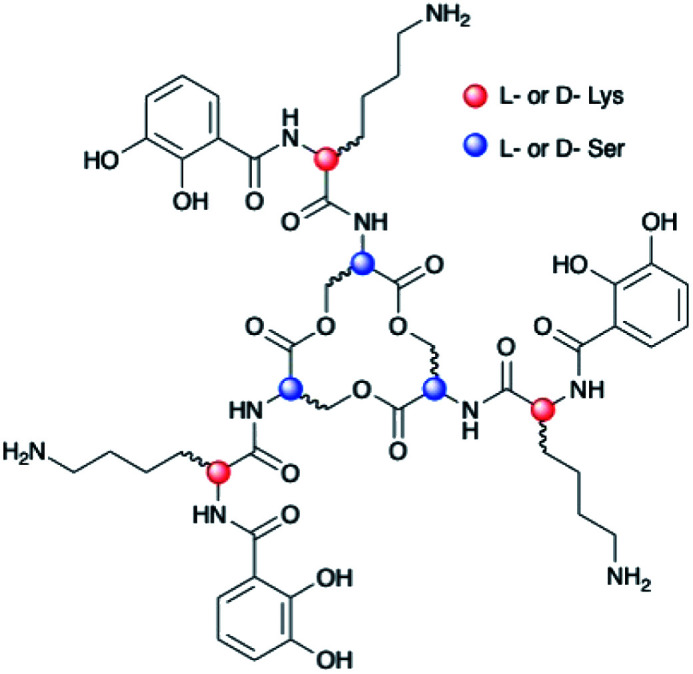
Diastereomers of (DHB^L/D^Lys^L/D^Ser)_3_.

To understand the factors controlling stereospecific Fe(iii) coordination of the expanded triscatechol–triserine siderophore CTC, (DHB^D^Lys^L^Ser)_3_, we synthesized the full suite of cyclic (DHB^L/D^Lys^L/D^Ser)_3_ diastereomers of CTC (Fig. 1). We report that circular dichroism (CD) spectroscopic measurements of the Fe(iii) complexes of these ligands allow the relationship between siderophore chirality and the configuration at the Fe(iii) site to be defined. Computational modeling of the ferric complexes reveals steric interactions between the Lys sidechains and the peptide backbone dictate the configurational preference. Fe(iii) complexation by each (DHB^L/D^Lys^L/D^Ser)_3_ diastereomer prompted microbial genome mining that led to the discovery of a new triscatechol siderophore, frederiksenibactin (FSB). FSB features ^L^Lys residues inserted between a linear triserine backbone and chelating DHB units. The genomics data, in combination with our spectroscopic investigation of isolated FSB, establish that it is a natural diastereomer of linear trichrysobactin. Circular dichroism (CD) spectra reveal that FSB and CTC coordinate Fe(iii) in opposing enantiomeric configuration, consistent with the setting of the handedness of Fe(iii) coordination by the stereochemistry at Lys.

## Results and discussion

### Chirality of Fe(iii)–[(DHB^L/D^Lys^L/D^Ser)_3_] diastereomers

Chiral triscatechol siderophores and synthetic analogs are capable of coordinating labile metal ions with a thermodynamic preference for a specific stereochemistry at the metal center.^8^ The presence of chirality at both the metal center and in the ligand renders the Δ and Λ stereoisomers diastereomeric and energetically inequivalent. To establish the relationship between the chirality at the amino acid adjacent to the catecholamide, the stereochemistry of the triserine macrolactone, and the stereochemistry at Fe(iii), we synthesized the four *C*_3_-symmetric cyclic diastereomers (DHB^L^Lys^L^Ser)_3_, (DHB^D^Lys^L^Ser)_3_, (DHB^L^Lys^D^Ser)_3_, and (DHB^D^Lys^D^Ser)_3_ (Scheme S1†) of which (DHB^D^Lys^L^Ser)_3_ is structurally identical to CTC. Well-established methodology to construct the cyclic triserine macrolactone (**1** in Scheme S1†),^9^ provided a convenient synthetic platform to access CTC and related diastereomers (Fig. S2–S6, Tables S1 and S2†). In the absence of crystallographic information, circular dichroism (CD) spectroscopy can provide information on the stereochemical configuration of optically-active metal complexes.^10^

**Fig. 2:**
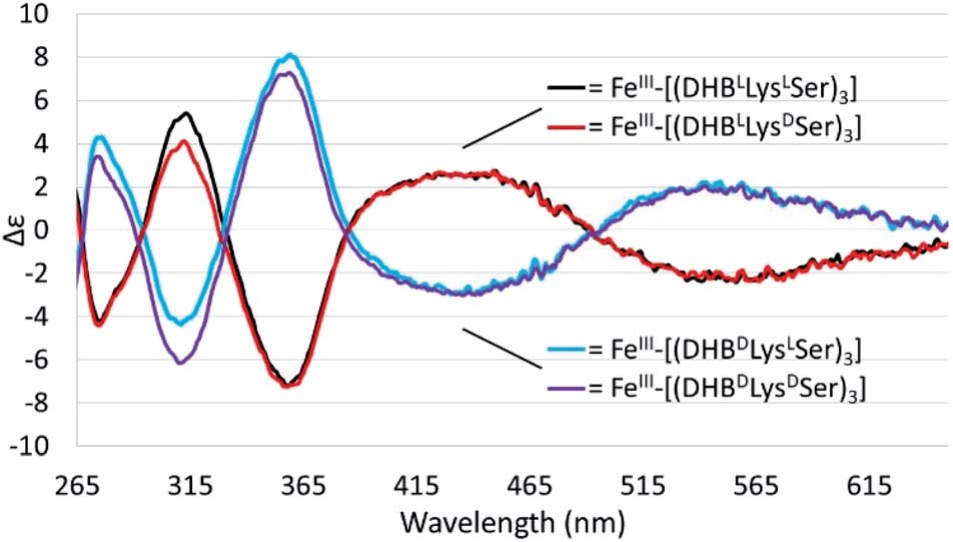
CD spectra of 40 mM solutions of the Fe(iii)–(DHB^L/D^Lys^L/D^Ser)_3_] complexes in citrate–phosphate buffer, pH 7.40.

As expected, enantiomeric pairs of ligands, such as (DHB^L^Lys^L^Ser)_3_ and (DHB^D^Lys^D^Ser)_3_, coordinate Fe(iii) with opposite handedness, as indicated by the CD spectra (Fig. 2). Two prominent CD bands at 435 nm and 545 nm arise from characteristic LMCT transitions and are therefore sensitive to the chirality at the iron center. When comparing diastereomeric ligands, we observed that the CD spectra of Fe(iii)–[(DHB^L^Lys^L^Ser)_3_] and Fe(iii)–[(DHB^L^Lys^D^Ser)_3_] are similar in both the sign and the intensity. The analogous correspondence was observed for the CD bands of Fe(iii)–[(DHB^D^Lys^L^Ser)_3_] (*i.e.*, CTC) and Fe(iii)–[(DHB^D^Lys^D^Ser)_3_] (Fig. 2, Table 1).‡‡Two prominent low-energy CD bands at 435 nm and 545 nm arise from characteristic LMCT transitions and are therefore sensitive to the chirality at the Fe(iii) center. The CD bands at 270 nm, 310 nm, and 360 nm are assigned to ligand-based transitions corresponding to the amide (270 nm) and ester (310 and 360 nm) carbonyls, in analogy to Fe(iii)–BB^3−^.^39^

**Table 1:** CD results of Fe(iii) complexes of FSB, CTC and related cyclic analogs^a^

Ligand	Cyclic (C) or linear (L)	Configuration	*λ*_max_ (nm)	Δ*ε* (M^−1^ cm^−1^)
Ent = (DHB^L^Ser)_3_ (ref. 3)	C	Δ	553	−2.2
BB = (DHB-Gly^L^Thr)_3_ (ref. 3)	C	Λ	545	+1.7
(DHB^L^Lys^L^Ser)_3_^b^	C	Δ	563	−2.3
(DHB^D^Lys^L^Ser)_3_^c^ (CTC)	C	Λ	559	+2.2
(DHB^L^Lys^D^Ser)_3_	C	Δ	561	−2.3
(DHB^D^Lys^D^Ser)_3_	C	Λ	558	+2.1
Frederiksenibactin (FSB) = *linear*-(DHB^L^Lys^L^Ser)_3_	L	Δ	556	−2.2

a 40 μM Fe(iii) complexes in citrate–phosphate buffer, pH 7.40.

b (DHB^L^Lys^L^Ser)_3_ is the cyclic analog of FSB.

c Synthetic (DHB^D^Lys^L^Ser)_3_ shown here is indistinguishable from CTC isolated from *D. chrysanthemi* EC16.

Comparison of the signs of the Cotton effects for the Fe(iii)–[(DHB^L/D^Lys^L/D^Ser)_3_] complexes with those of Fe(iii)–Ent^3−^ and Fe(iii)–BB^3−^, for which the chirality at the metal center is known, allows the configuration of the new complexes to be determined. (DHB^L^Lys^L^Ser)_3_ and (DHB^L^Lys^D^Ser)_3_ both form Δ complexes, whereas (DHB^D^Lys^D^Ser)_3_ and (DHB^D^Lys^L^Ser)_3_ both form Λ complexes. These results suggest that the handedness of metal-ion chelation is set by the chirality of the Lys unit, and not the chirality of the triserine macrolactone. In comparison, the Δ configuration of Fe(iii)–Ent^3−^ and Λ configuration of Fe(iii)–enantioEnt^3−^ has been attributed to nonbonding interactions within the chiral triserine macrolactone.^11–13^

### Computational modeling

**Fig. 3:**
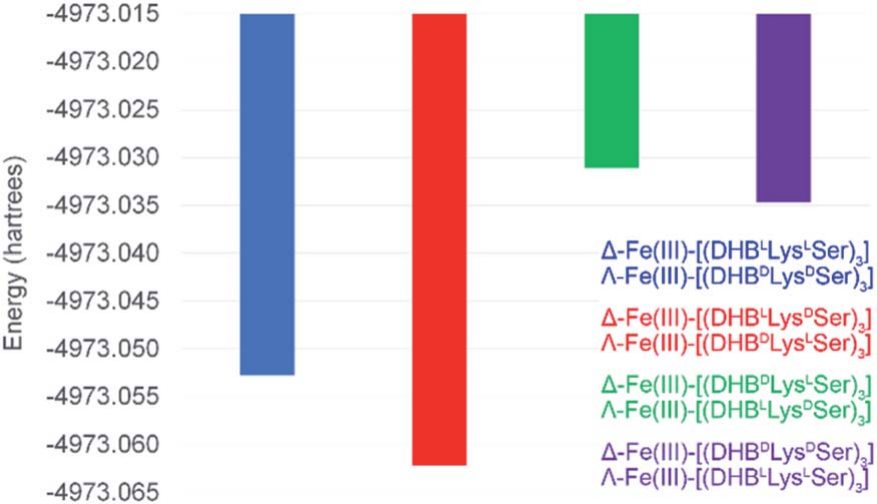
Energies for the optimized structures of the eight Fe(iii)–[(DHB^L/D^Lys^L/D^Ser)_3_] diastereomers.

To better understand the mechanism by which amino acid chirality dictates the configurational preferences of the Fe(iii)–[(DHB^L/D^Lys^L/D^Ser)_3_] complexes, the structures and energies of the four enantiomeric pairs of diastereomers were optimized computationally (PBE0/6-311++G(d,p)) (Fig. 3).

Comparing the energies of the Fe(iii) complexes of a given ligand with different handedness, we observe complete agreement with the CD spectroscopic results. For example, the energy of Λ-Fe(iii)–[(DHB^D^Lys^L^Ser)_3_] is lower than that of Δ-Fe(iii)–[(DHB^D^Lys^L^Ser)_3_], consistent with the formation of the Λ complex in aqueous solution (Fig. 2).

**Fig. 4:**
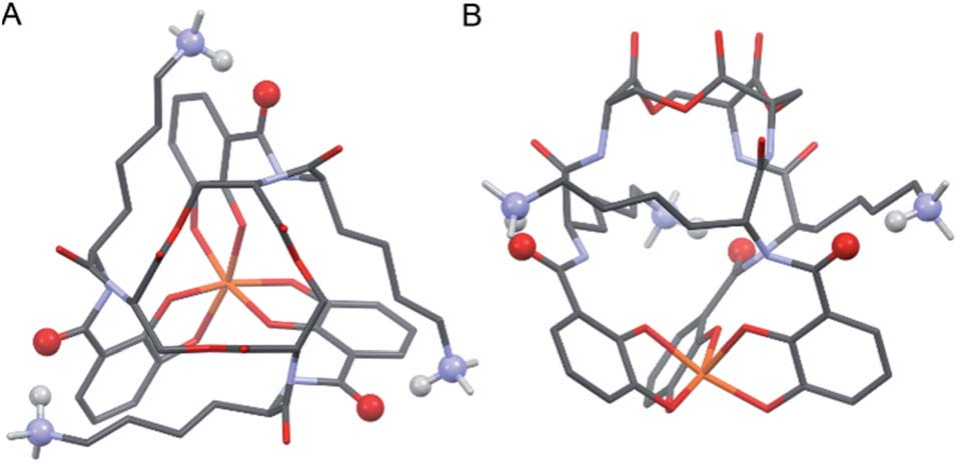
Stick representations of the computationally (PBE0/6-311++G(d,p)) optimized structure of Λ-Fe(iii)–[(DHB^D^Lys^L^Ser)_3_] from the top (left) and side (right). H atoms removed for clarity except on Lys NH_3_ units. Color code: Fe orange, O red, N blue, C grey, H white. The atoms engaging in Lys-DHB intramolecular hydrogen bonding are shown as balls.

Insight into the origin of the differential stabilities of the Fe(iii)–[(DHB^L/D^Lys^L/D^Ser)_3_] complexes comes directly from the optimized geometries (Fig. 4). In this molecular framework, the Lys sidechains are able to wrap around the complex so as to allow each terminal ammonium group to hydrogen-bond with the carbonyl of the DHB unit of an adjacent arm. We observe, however, that this interaction is present in all of the optimized geometries, preferred and non-preferred.

**Fig. 5:**
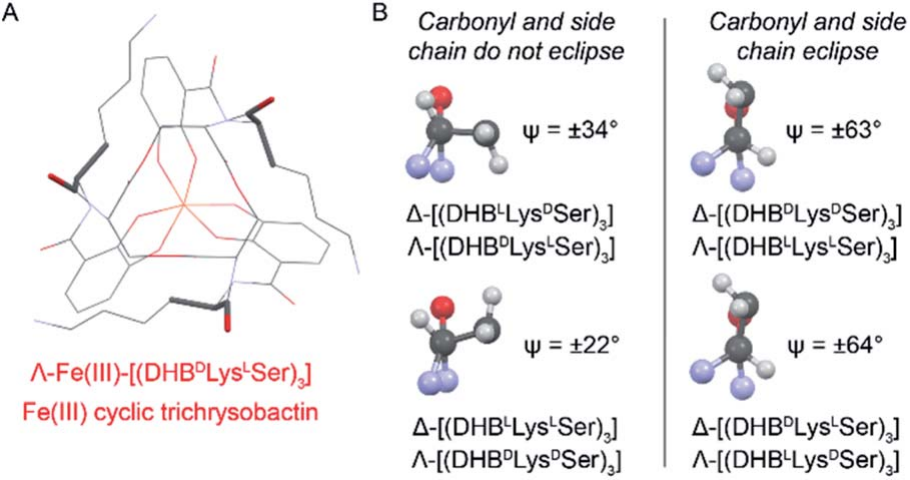
Left: Line diagram of Λ-Fe(iii)–[(DHB^D^Lys^L^Ser)_3_] from the top with the carbonyl and first Lys side chain length shown as sticks. Right: Views along the C_α_–C_carbonyl_ bonds of Lys residues from the indicated Fe(iii) complexes. *Ψ* values are averaged across the three present in each structure. Positive and negative *Ψ* values correspond to the compounds with ^D^Lys and ^L^Lys, respectively.

Closer analysis revealed that the prime influence of the Lys residue chirality is the impact that it has on *ψ* (N–C_carbonyl_–C_α_–N torsion angle). It is well established that certain values of *ψ* are unfavorable for polypeptides, contributing, for example, to the characteristic distribution of protein dihedral angles in Ramachandran plots. Specifically, favorable *ψ* values are those that prevent the amino acid side chain from eclipsing the adjacent carbonyl.^14^ In the Fe(iii)–[(DHB^L/D^Lys^L/D^Ser)_3_] complexes, combination of either Δ configuration at Fe(iii) and ^D^Lys, or Λ and ^L^Lys, produce *ψ* angles near ±60°, which introduces a steric clash between the carbonyl O atom and the Lys side chain (Fig. 5). In contrast, the combination of Λ and ^D^Lys, as occurs in CTC, produces no such clash.

It is noteworthy that, of all the diastereomeric combinations of metal chelation handedness and amino acid chirality, our calculations predict that the most stable structures are those assumed by Δ-Fe(iii)–[(DHB^L^Lys^D^Ser)_3_] and its enantiomer Λ-Fe(iii)–[(DHB^D^Lys^L^Ser)_3_]. Organisms have adopted this stability by using (DHB^D^Lys^L^Ser)_3_, which is the siderophore CTC, for iron acquisition. The fact that the other diastereomers that we investigated also form Fe(iii) complexes gives rise to the question of whether they too might be used biologically.

### Genomic screen for catechol-based siderophores

**Fig. 6:**
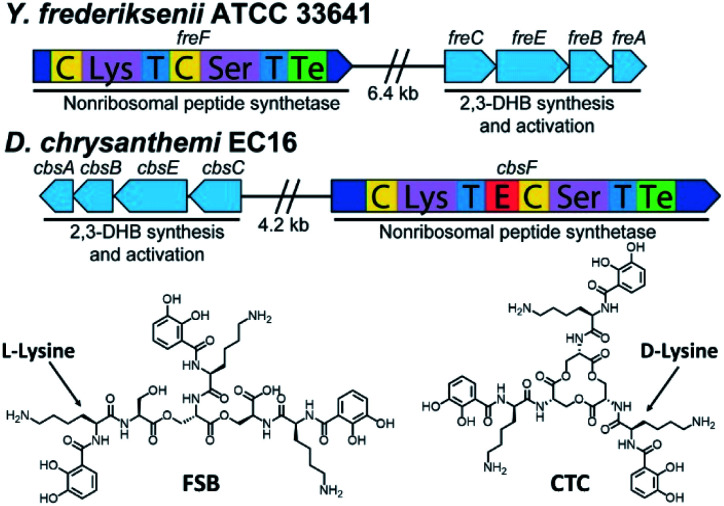
Biosynthetic gene clusters and structures of frederiksenibactin, FSB (*Y. frederiksenii* ATCC 33641) and cyclic trichrysobactin, CTC (*D. chrysanthemi* EC16).

Inspired by the discovery of other naturally occurring siderophores with d- and l-amino acids – that is, trivanchrobactin (^D^Arg), and turnerbactin (^L^Orn) – we initiated a search for biosynthetic gene clusters (BGCs) encoding diastereomers of CTC. The biosynthesis of chrysobactin (*i.e.*, DHB^D^Lys^L^Ser) in *D. dadantii* 3937 requires genes encoding 2,3-DHB synthesis, as well as the non-ribosomal peptide synthetase (NRPS) CbsF with an epimerization, E, domain to convert ^L^Lys to ^D^Lys.^15,16^ In contrast to *D. dadantii* 3937, the plant pathogen *D. chrysanthemi* EC16 produces not only the monocatechol chrysobactin, but also the triscatechol macrolactone CTC. We found that the genome of the *D. chrysanthemi* EC16 contains a BGC homologous to the *cbs* locus of *D. dadantii* 3937 (genome sequence reported herein; Tables S3 and S4†). Genome mining revealed similar but distinct BGCs in several *Yersinia* genomes, including the BGC *freABCEF* of opportunistic pathogen *Yersinia frederiksenii* ATCC 33641 (Tables S5 and S6†). The *fre* locus contains genes encoding 2,3-DHB synthesis, as well as the NRPS FreF with adenylation domains selecting for ^L^Lys and ^L^Ser. However, FreF lacks an E domain, implicating biosynthesis of a siderophore comprised of DHB^L^Lys^L^Ser units (Fig. 6).

### Frederiksenibactin and cyclic trichrysobactin siderophores

Siderophores from *Y. frederiksenii* ATCC 33641 were extracted and purified from a low-iron culture (Fig. S7†). Three compounds with *m*/*z* of 370.17, 721.31 and 1072.44 were identified by UPLC-ESIMS. These signals are equivalent to the [M + H]^+^ signals for chrysobactin, linear dichrysobactin, and linear trichrysobactin, which are DHB-^D^Lys-^L^Ser, *linear*-(DHB-^D^Lys-^L^Ser)_2_, and *linear*-(DHB-^D^Lys-^L^Ser)_3_, respectively.^5^ In contrast to *D. chrysanthemi* EC16, which produces trichrysobactin in both cyclic and linear forms,^5^ we have only been able to detect a linear triscatechol siderophore (*m*/*z* 1072.44; Fig. S8†) in the culture supernatant of *Y. frederiksenii* ATCC 33641. We have named this new siderophore frederiksenibactin (FSB). We note that the related triscatechol siderophores trivanchrobactin and turnerbactin are also linear and that their cyclic forms have not been detected in biological systems.^6,7^

Marfey's analysis^17^ establishes the presence of ^L^Lys and ^L^Ser in FSB, consistent with the genomic prediction (Fig. S9†). The proposed structure of FSB was confirmed by ^1^H and ^13^C NMR spectroscopic data, which were assigned through ^1^H–^1^H COSY, ^1^H–^13^C HSQC, and ^1^H–^13^C HMBC NMR data (Fig. S10–S14†). While the NMR spectral data of FSB (Table S7†) are similar to those of CTC, several features confirm the mass spectrometric results indicating that FSB is a linear compound. Specifically, the three Ser residues are inequivalent (Fig. S11†). The Ser methylene protons involved in the backbone ester linkages, C16/C16′, at 4.25–4.46 ppm are shifted significantly downfield relative to the corresponding protons on C16′′ at 3.67 ppm and 3.78 ppm, which are adjacent to the unmodified Ser hydroxyl group. Additionally, the protons on the three methine carbons (C15, 4.59 ppm; C15′, 4.69 ppm; C15′′, 4.41 ppm) are inequivalent, as are the protons on the chiral methine carbons derived from Lys (C9, C9′ and C9′′, 4.50–4.65 ppm). The ^1^H NMR spectrum of FSB is consistent with related asymmetric linear triscatechol siderophores trivanchrobactin^6^ and turnerbactin.^7^ Thus, FSB is a novel siderophore and a natural diastereomer of linear trichrysobactin.

### Chirality of Fe(iii)–FSB and Fe(iii)–CTC

**Fig. 7:**
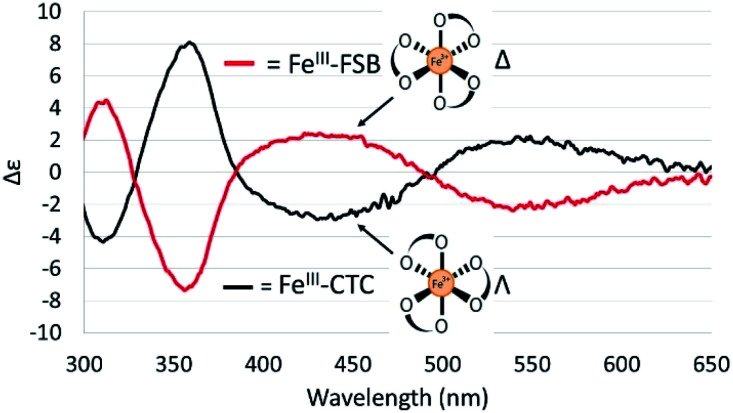
CD spectra of 40 μM Fe(iii)–FSB and 40 μM Fe(iii)–CTC in citrate–phosphate buffer (pH 7.40).

The CD spectra of Fe(iii)–CTC and Fe(iii)–FSB (Fig. 7) appear as near mirror-images of each other, indicating an opposite configurational preference around iron. Through comparison to the CD spectra of Fe(iii)–Ent^3−^, Fe(iii)–BB, and the Fe(iii)–[(DHB^L/D^Lys^L/D^Ser)_3_] complexes (Table 1)^3,4^ Fe(iii)–FSB is assigned a Δ configuration. The comparison of Fe(iii)–FSB to Fe(iii)–[(DHB^L^Lys^L^Ser)_3_] (Fig. 2, Table 1) also establishes that linearization of the trilactone does not significantly affect the configuration of the ferric complex. Earlier work revealed that linearization of Ent also does not invert its overall configurational preference, however, a small fraction of the Λ enantiomer is formed.^11,13^ Our earlier work with the cyclic Fe(iii)–[(DHB^L/D^Lys^L/D^Ser)_3_] complexes suggests that the opposing chirality observed for ferric complexes of FSB and CTC is likely due to the stereochemistry of the Lys residue adjacent to the catecholamide and not due to the linear or cyclic nature of the triserine backbone.

### Fe(iii) exchange between FSB and CTC

**Fig. 8:**
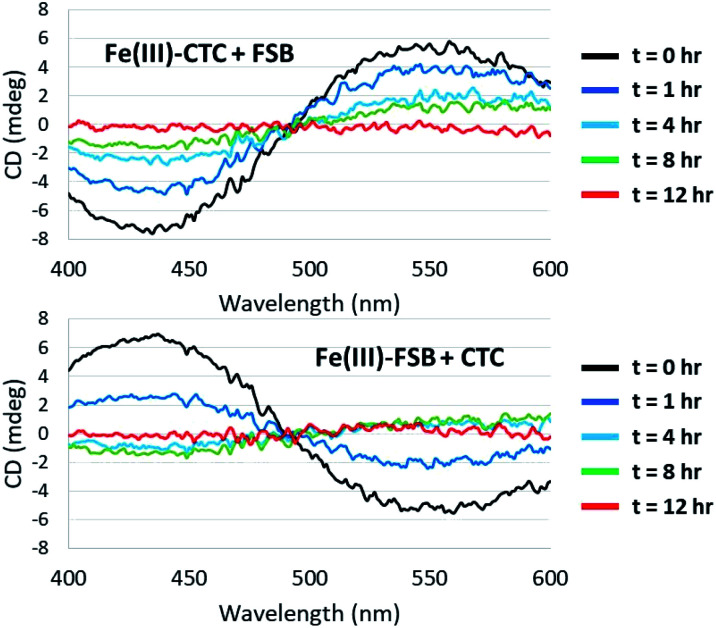
Equilibration of Fe(iii)–CTC and Fe(iii)–FSB with FSB and CTC, respectively. (Top) Reaction of 100 μM Fe(iii)–CTC with 100 μM FSB in 50 mM citrate–phosphate, pH 7.40. (Bottom) Reaction of 100 μM Fe(iii)–FSB with 100 μM CTC in 50 mM citrate–phosphate, pH 7.40.

Surprisingly little is known about the exchange of Fe(iii) among triscatechol siderophores. CD spectroscopy is uniquely poised to monitor Fe(iii) exchange between optically-active siderophores. The intensity of the Λ-Fe(iii)–CTC CD bands decrease upon addition of equimolar FSB as a result of formation of nearly equimolar Λ-Fe(iii)–CTC and Δ-Fe(iii)–FSB (Fig. 8A). Moreover the equivalent equilibration approached from reaction of Δ-Fe(iii)–FSB with CTC is also observed (Fig. 8B). Interestingly, a weak negative band at 435 nm and a weak positive band at 550 nm are formed after four hours of equilibration (Fig. 8B), suggestive of a slight preference favoring formation of Fe(iii)–CTC over Fe(iii)–FSB, consistent with the increased stability constant of macrocyclic ligands.^13^ The intensity of this band diminishes upon further equilibration, potentially due to hydrolysis of the labile macrolactone. Under neutral pH conditions with 100 μM Fe(iii)–CTC and 100 μM FSB, the magnitude of the CD signal decreases within hours of mixing, indicating that exchange occurs on a relatively short, biologically relevant time scale.

## Conclusions

In sum, BGCs encoding synthesis of the triscatechol siderophores CTC and FSB were identified and the structure of FSB was elucidated. The opposing configurations of Δ-Fe(iii)–FSB and Λ-Fe(iii)–CTC are established by the stereochemistry at Lys. The most stable configuration by computational modeling is Λ-Fe(iii)–[(DHB^D^Lys^L^Ser)_3_], which, strikingly, has been adopted for microbial iron acquisition as Fe(iii)–CTC. The next most stable conformation corresponds to the cyclic form of FSB, Δ-Fe(iii)–[(DHB^L^Lys^L^Ser)_3_], raising questions about the predicted low energy conformations of the corresponding linear triscatechol siderophores.

The suite of cyclic and linear (DHB^L/D^Lys^L/D^Ser)_3_ siderophores and analogs raises further significant questions regarding the effect of a mismatched Δ- and Λ-Fe(iii) configuration on microbial iron uptake and growth. For example, discrimination at the outer membrane receptor protein could prevent uptake of the wrong Fe(iii)–enantiomer as has been observed with Fe(iii) complexes of pyochelin and enantiopyochelin.^18–20^ If iron uptake is insensitive to the Fe(iii)–enantiomer chirality, discrimination could still occur at other points including the iron-release process, as is observed in *Bacillus subtilis* in the Fes-catalyzed hydrolysis of the macrolactone of Fe(enantioEnt)^3−^ which is required for release of iron.^1^ Additionally, it may be possible for the relevant siderophore-interacting proteins to invert the configuration of a mismatched Fe(iii)–siderophore complex upon binding, as has been observed for the periplasmic binding protein CeuE of *Campylobacter jejuni*.^21–23^

Siderophores are primarily extracellular metabolites and facile Fe(iii) exchange observed between triscatechol siderophores is likely of biological consequence within complex microbial communities. Certainly, the rate of release of the newly synthesized apo siderophores during growth of *Y. frederiksenii* ATCC 33641 and *D. chrysanthemi* EC16, which is occurring over the time scale of hours to days, could be exchanging Fe(iii) within hours with other triscatechol siderophores, as evinced by the CD results (Fig. 8). In fact Fe(iii) exchange between the triscatecholate siderophores is orders of magnitude faster than Fe(iii) exchange between hydroxamate siderophores or between hydroxamate and catecholate siderophores.^24^

Experiments addressing the questions raised above are in progress, as well as the question of whether BGCs encoding the synthesis of the diastereomers of trivanchrobactin, (DHB^D/L^Arg^L/D^Ser)_3_, and turnerbactin, (DHB^L/D^Orn^L/D^Ser)_3_, are present in microbial genomes. The discovery of frederiksenibactin and its relationship to CTC exemplifies the structural variability of microbial siderophores and provides a natural system to determine the significance of chirality within siderophore-mediated microbial iron-uptake pathways.

## Experimental

### General experimental procedures

UV-visible absorbance and circular dichroism spectroscopy were measured on an Agilent Cary 300 UV Vis spectrophotometer and a Jasco J-1500 CD spectrophotometer, respectively. ^13^C NMR spectroscopy was performed on a Bruker Advanced Neo 500 MHz spectrometer equipped with a prodigy cryoprobe at RT. All ^1^H, COSY, HMBC, HSQC NMR spectroscopy was performed on a Varian Unity 600 MHz spectrometer at RT. Chemical shifts were referenced through residual solvent peaks [^1^H (DMSO-*d*_6_) 2.50 ppm, ^13^C (DMSO-*d*_6_) 39.51 ppm]. Mass spectrometry analysis of *Y. frederiksenii* ATCC 33641 supernatant extracts and purified FSB was carried out on a Waters Xevo G2-XS QToF with positive mode electrospray ionization coupled to an ACQUITY UPLC H-Class system with a waters BEH C18 column. *Y. frederiksenii* ATCC 33641 culture extracts were analyzed using a linear gradient of 0–30% CH_3_CN (+0.1% formic acid) in ddH_2_O (+0.1% formic acid) over 10 min. For MS/MS analysis, a collision energy of 15 eV was employed. HR-ESIMS analysis of synthetic compounds was carried out on a Waters LCT Premier ESI TOF introduced into the ESI by direct infusion *via* a syringe pump.

### General synthetic procedures

All reactions performed under an argon atmosphere were carried out using a high-vacuum line, standard Schlenk techniques, and dry solvents. DMF, DCM, and DMSO-*d*_6_ were stored over 3 Å molecular sieves for at least 72 h prior to use. *N*,*N*′-Diisopropylethylamine (DIPEA) was purified by distillation over ninhydrin (×3) and was subsequently stored over 3 Å molecular sieves. *N*_α_-Boc-*N*_ε_-Cbz-l-lysine and *N*_α_-Boc-*N*_ε_-Cbz-d-lysine were acquired from Bachem. All other reagents (including those used for Marfey's analysis) were purchased from Sigma-Aldrich.

### Synthesis of the cyclic (DHB^L/D^Lys^L/D^Ser)_3_ diastereomers

Established peptide coupling methodology^25^ was employed to construct the two key amide bonds in **4** (Scheme S1†). Reaction of chiral triamine **1** with HATU (3 eq.), Boc–Lys(Z)–OH (3 eq.), and DIPEA (9 eq.) cleanly affords intermediate **2** (Step a, Scheme S1†). Removal of the *N*_α_-Boc protecting groups (step b, Scheme S1†) and subsequent coupling to benzyl-protected 2,3-dihydroxybenzoic acid (step c, Scheme S1†) yields **3** in an 81% yield over two steps. Global deprotection by hydrogenolysis over 10% Pd/C (step d, Scheme S1†) yields (DHB^L/D^Lys^L/D^Ser)_3_, (**4**) as an enantiopure product. Initial synthetic efforts in which the direction of peptide coupling was reversed were highly susceptible to epimerization at the Lys stereocenter, consistent with the observed chiral instability of *N*_α_-acylated amino acids upon activation as a HOBT or HOAT ester.^26^*N*_α_-Boc–*N*_ε_-Cbz-l-lysine was substituted for *N*_α_-Boc-*N*_ε_-Cbz-d-lysine in the synthesis of (DHB^D^Lys^L^Ser)_3_ and (DHB^D^Lys^D^Ser)_3_. *N*-Trityl-l-serine was substituted for *N*-trityl-d-serine in the synthesis of **1** to yield (DHB^L^Lys^D^Ser)_3_ and (DHB^D^Lys^D^Ser)_3_.

#### Synthesis of *N*,*N*′,*N*′′-tris[*N*_α_-Boc-*N*_ε_-Cbz-l-lysinyl]cyclotri-l-seryl trilactone, **2**

*N*_α_-Boc-*N*_ε_-Cbz-l-lysine (502 mg, 1.32 mmol) was dissolved in 10 mL of dry DMF under an argon atmosphere and cooled in an ice bath. HATU (502 mg, 1.32 mmol) and DIPEA (836 μL, 4.8 mmol) were added at 0 °C and the flask was subsequently taken out of the ice bath and stirred for 3 min. Triserine trilactone hydrochloride (148.5 mg, 0.4 mmol), prepared according to literature procedure,^27^ was added as a solid to the flask and the reaction was stirred overnight at RT. The solvent was removed *in vacuo* and the crude reaction mixture was brought up in DCM and rinsed quickly with 1 M HCl (30 mL, ×3) and brine (30 mL). The organic layer was concentrated and then loaded onto a silica column. Purification by flash chromatography using a gradient of 2–4% MeOH in DCM afforded **2** as a colorless solid. (76% yield). ^1^H NMR (DMSO-*d*_6_, 25 °C): *δ* = 1.20–1.60 (m, 45H; CH_2_, CH_3_), 2.96 (m, 6H; CH_2_), 3.91 (td, *J* = 8.5, 4.5, 3H; CH), 4.10 (dd, *J* = 11.0, 4.5, 3H; CH_2_), 4.36 (t, *J* = 10.1, 3H; CH_2_), 4.59 (m, 3H; CH), 5.00 (s, 6H; CH_2_), 6.85 (d, *J* = 7.9, 3H; NH), 7.21 (t, *J* = 5.7, 3H; NH), 7.28–7.38 (m, 15H; Ar-H), 8.34 (d, *J* = 7.4; 3H) ppm. ^13^C NMR (DMSO-*d*_6_, 25 °C): *δ* = 22.7, 28.2, 29.1, 31.4, 38.2, 50.6, 54.0, 63.1, 65.1, 78.1, 127.7, 128.3, 137.3, 155.4, 156.0, 169.5, 172.6 ppm. HRMS (ESI) *m*/*z* calcd for C_66_H_93_N_9_O_21_ + Na^+^: 1370.6384 [M + Na]^+^; found: 1370.6362.

#### Synthesis of *N*,*N*′,*N*′′-tris[*N*_α_-2,3-di(benzyloxy)benzoyl-*N*_ε_-Cbz-l-lysinyl]cyclotri-l-seryl trilactone (BnDHB^L^Lys^L^Ser)_3_, **3**

Compound **2** (404.6 mg, 0.3 mmol) was added to a dry flask under argon and dissolved in 6 mL dry DCM. The flask was cooled in an ice bath and 4 mL of TFA were added. After stirring for 1.5 h at RT, full deprotection of the boc groups was observed by TLC. Volatiles were removed *in vacuo* and the pale yellow oil was brought up in 5 mL of dry DMF. In a separate flask, 2,3-dibenzyloxybenzoic acid (341 mg, 0.99 mmol), HATU (376 mg, 0.99 mmol), and DIPEA (627 μL, 3.6 mmol) were added to 5 mL of dry DMF under an argon atmosphere and stirred for 3 min at RT. The contents of the first flask were then transferred to the reaction mixture *via* syringe and the reaction was left to stir overnight at RT. The reaction mixture was concentrated, loaded onto a silica column, and then purified by flash chromatography using a gradient of 1–3% MeOH in DCM. Fractions were combined and concentrated to yield **3** as a white solid. (81% yield over 2 steps) ^1^H NMR (DMSO-*d*_6_, 25 °C): *δ* = 1.28 (m, 6H; CH_2_), 1.33 (m, 6H; CH_2_), 1.47 (m, 3H; CH_2_), 1.61 (m, 3H; CH_2_), 2.93 (m, 6H; CH_2_), 4.13 (m, 3H; CH), 4.38 (t, *J* = 10.3, 3H; CH_2_), 4.49 (td, *J* = 8.3, 5.2, 3H; CH_2_), 4.64 (m, 3H; CH), 4.96 (s, 6H; CH_2_), 4.99 (d, *J* = 10.6, 3H; CH), 5.08 (d, *J* = 10.6, 3H; CH_2_), 5.20 (s, 6H; CH_2_), 7.15 (m, 6H; NH, Ar-H), 7.20–7.43 (m, 45H; Ar-H), 7.50 (m, 6H; Ar-H), 8.43 (d, *J* = 7.7, 3H; NH), 8.63 (d, *J* = 7.1, 3H; NH) ppm. ^13^C NMR (DMSO-*d*_6_, 25 °C): *δ* = 22.5, 29.1, 31.9, 38.2, 50.7, 52.6, 65.1, 70.3, 75.1, 116.5, 121.4, 124.2, 127.7, 127.9, 128.0, 128.1, 128.3, 128.4, 128.9, 136.6, 136.7, 137.2, 145.6, 151.6, 156.0, 162.3, 164.9, 169.3, 171.8 ppm. HRMS (ESI) *m*/*z* calcd for C_114_H_117_N_9_O_24_+2Na^+^: 1020.9004 [M + 2Na]^2+^; found: 1020.9017.

#### *N*,*N*′,*N*′′-Tris[2,3-dihydroxybenzoyl-l-lysinyl]cyclotri-l-seryl trilactone (DHB^L^Lys^L^Ser)_3_, **4**

Compound **3** (399.5 mg, 0.2 mmol) was dissolved in 10 mL of 60% THF (aq.) + 0.5% acetic acid under an atmosphere of argon. 10% Pd/C (100 mg) was carefully added, and a balloon of hydrogen attached to a three-way flushing adapter was fitted to the round bottom. The atmosphere was evacuated and back-filled with hydrogen four times and stirred under an atmosphere of hydrogen for 24 h at RT. The catalyst was then filtered off, rinsed with 25 mL of DMF, and concentrated to yield a dark-red oil. The crude reaction, deemed mostly pure by NMR, was further purified by semi-preparative HPLC on a YMC-Actus 20 × 250 mm C18 ODS-AQ column using a linear gradient of 15% MeOH in ddH_2_O (+0.1% trifluoroacetic acid) to 40% MeOH in ddH_2_O (+0.1% trifluoroacetic acid) over 25 min. HPLC fractions were concentrated and subsequently lyophilized to yield **4** as a white solid. (65% yield) ^1^H NMR (DMSO-*d*_6_, 25 °C): *δ* = 1.37 (m, 6H; CH_2_), 1.55 (m, 6H; CH_2_), 1.75 (m, 6H; CH_2_), 2.77 (m, 6H; CH_2_), 4.14 (dd, *J* = 10.8, 6.2, 3H; CH_2_), 4.43 (t, *J* = 10.6, 3H; CH_2_), 4.53 (td, *J* = 8.5, 4.8, 3H; CH), 4.64 (ddd, *J* = 10.1, 7.2, 4.7, 3H; CH), 6.70 (t, *J* = 7.9, 3H; Ar-H), 6.95 (d, *J* = 7.8, 3H; Ar-H), 7.40 (d, *J* = 8.2, 3H; Ar-H), 7.81 (s, 9H; NH_3_), 8.77 (d, *J* = 7.2, 3H; NH), 8.80 (d, *J* = 7.6, 3H; NH), 9.40 (s, < 3H; OH), 11.91 (s, 3H; OH) ppm. ^13^C NMR (DMSO-*d*_6_, 25 °C): *δ* = 22.5, 26.7, 31.1, 38.7, 50.8, 52.6, 63.1, 115.9, 118.2, 118.4, 118.9, 146.1, 148.6, 169.5, 171.6 ppm. HRMS (ESI) *m*/*z* calcd for C_48_H_63_N_9_O_18_ + 2H^+^: 527.7224 [M + 2H]^2+^; found: 527.7213.

### Amino acid analysis of frederiksenibactin and synthetic cyclic (DHB^L/D^Lys^L/D^Ser)_3_ analogs by Marfey's method

(DHB^L/D^Lys^L/D^Ser)_3_ or FSB (2 mg) was dissolved in 2 M HCl and heated at 110 °C in a sealed glass ampule under argon for 24 h. The hydrolysis mixture was evaporated to dryness under a stream of air and redissolved in 100 μL ddH_2_O. 1-Fluoro-2,4-dinitrophenyl-l-alanine amide (FDAA, 1 M in acetone, 150 μL) and NaHCO_3_ (1 M, 20 μL) were added and the solution was briefly vortexed and placed on a heating block (40 °C) for 1 h. 10 μL of 2 M HCl was then added to quench the reaction and solutions were stored at −20 °C in the dark prior to analysis. Amino acid standards were derivatized according to the same procedure. Derivatized hydrolysis products of FSB were separated by HPLC on a YMC 4.6 × 250 mm C18-AQ column using a gradient from 10% CH_3_CN in ddH_2_O (0.05% trifluoroacetic acid) to 40% CH_3_CN in ddH_2_O (0.05% trifluoroacetic acid) over 60 min. Derivatized hydrolysis products of (DHB^L/D^Lys^L/D^Ser)_3_ were separated by HPLC on a YMC 4.6 × 250 mm C18-A column using a gradient from 10% CH_3_CN in TEAP buffer (50 mM, pH 3.00) to 40% CH_3_CN in TEAP buffer over 60 min. Derivatized hydrolysis products were co-injected with derivatized amino acid standards to determine the constituent amino acids of FSB and to determine the extent of epimerization during synthesis of synthetic (DHB^L/D^Lys^L/D^Ser)_3_. Three peaks corresponding to FDAA-derivatized lysine were observed, corresponding to products derivatized at either the α-amine, ε-amine, or both amines. FDAA-derivatized ^D^Ser co-eluted with ^L^Lys and ^D^Lys derivatized at the ε-amine under the conditions used for Marfey's analysis of FSB (YMC C18-AQ column).

### Preparation of Fe(iii)-complexes and circular dichroism spectroscopy

Fe(iii)-complexes of the (DHB^L/D^Lys^L/D^Ser)_3_ diastereomers and FSB for CD spectroscopy were prepared in citrate–phosphate buffer (50 mM, pH 7.40) by mixing a solution of FeCl_3_ [2.45 mM, 0.1 M HCl (aq)] with 1.0 equivalent of the desired apo-ligand. Formation of the Fe(iii)-complex was tracked by UV-visible spectroscopy by observing the absorbance at 498 nm. The resulting solution was equilibrated for 30 min in the dark prior to analysis by CD spectroscopy.

Full CD spectra were acquired using the following parameters: 4 s D.I.T., 1 nm bandwidth, 50 nm s^−1^ scanning speed, with 3 accumulations. Fe(iii) exchange assays were performed by preparing pre-equilibrated Fe(iii)-complexes of either FSB or CTC as described above. At time *t* = 0, an equimolar amount of the opposing apo-ligand was added to the Fe(iii)-complex and the resulting solution was gently vortexed. CD spectra were acquired as a single accumulation at 20 min intervals using the following parameters: 400–600 nm; 2 s D.I.T., 1 nm bandwidth, and 100 nm s^−1^ scan speed.

### Computational modeling

Electronic structure calculations were performed using Gaussian 16.^28^ The structures of the following four complexes were optimized: Δ-Fe(iii)–[(DHB^L^Lys^L^Ser)_3_], Δ-Fe(iii)–[(DHB^L^Lys^D^Ser)_3_], Δ-Fe(iii)–[(DHB^D^Lys^L^Ser)_3_], and Δ-Fe(iii)–[(DHB^D^Lys^D^Ser)_3_]. Note that the structures of the corresponding Λ isomers were not optimized, because each is an enantiomer of one of the four Δ complexes listed above, and therefore energetically equivalent. The input geometries were generated manually. The Fe(iii) centers were treated as high-spin (*S* = 5/2) and the Lys residues were protonated to afford neutral complexes (*z* = 0). Optimizations were performed at the PBE0/6-311++G(d,p) level of theory with Grimme's D3 empirical dispersion correction and tight convergence criteria.^29–32^

Implicit aqueous solvation was included using a conductor-like polarizable continuum model (CPCM). The energy values presented in Fig. 3 are electronic energies that have not been zero-point corrected. Optimized coordinates are collected in Tables S8–S11.† Representations of Λ-Fe(iii)–[(DHB^D^Lys^L^Ser)_3_] (*i.e.*, Fe(iii)–CTC) were generated by inverting the optimized coordinates of Δ-Fe(iii)–[(DHB^L^Lys^D^Ser)_3_]. Geometric analyses were performed using Mercury.^33^

### Genome sequence of *Dickeya chrysanthemi* EC16 (ATCC 11662)

*Dickeya chrysanthemi* EC16 (ATCC 11662) was obtained from the ATCC and maintained on Difco Luria–Bertani (LB) agar plates at 30 °C. A liquid LB culture was inoculated from a single colony and incubated for 18 h at 30 °C and 180 rpm. Genomic DNA was extracted using the DNeasy Blood and Tissue Kit (Qiagen) according to the manufacturer's instructions for Gram-negative bacteria. Extracted DNA was quantified by a Qubit 2.0 fluorometer (Invitrogen). Library preparation and sequencing were performed by the Microbial Genome Sequencing Center (Pittsburgh, PA): paired-end libraries were prepared according to Baym *et al.*^34^ and sequenced on the NextSeq 550 platform (Illumina), generating 4 232 664 pairs of 2 × 150 bp reads.

Read quality was assessed with FastQC v0.11.9 (https://github.com/s-andrews/FastQC). Low-quality reads were trimmed with trimmomatic using the settings “LEADING:10 TRAILING:10 SLIDINGWINDOW:4:20 MINLEN:80”. Trimmed reads were assembled using SPAdes v3.14.1 with the flags “--isolate -k 21,33,55,77” recommended for bacterial isolates.^35^ Scaffold quality was assessed by QUAST v5.1.0rc1.^36^ Scaffolds over 500 bp in length were retained and quality was assessed by QUAST. The final assemblies were annotated using the NCBI Prokaryotic Genome Annotation Pipeline v5.0.^37^ Default parameters were used, except where otherwise noted. Assembly statistics are given in Table S3.† Taxonomic classification was determined by comparing average-nucleotide identity (ANI) against type strain Genbank sequences using OrthoANIu.^38^ By this metric, the previous assignment of strain EC16 as *D. chrysanthemi* is strongly supported, with a 99.97% ANI with *D. chrysanthemi* NCPPB 402^T^.

### Bacterial growth and siderophore isolation

*Yersinia frederiksenii* ATCC 33641, obtained from the American Type Culture Collection (ATCC), was cultured on Difco Luria Bertani (LB) Miller (BD biosciences) medium plates. A single colony of *Y. frederiksenii* ATCC 33641 was inoculated into 50 mL of Difco LB Miller (BD biosciences) media and grown overnight at 30 °C, shaking at 180 rpm. A portion of the overnight culture (5 mL) was then inoculated into low-iron minimal media (2 L, pH 7.0) containing sodium succinate (4 g L^−1^), K_2_HPO_4_ (6 g L^−1^), KH_2_PO_4_ (3 g L^−1^), NH_4_Cl (1 g L^−1^), CaCl_2_·2H_2_O (20 mg L^−1^), and MgSO_4_·7H_2_O (200 mg L^−1^) in an acid-washed 4 L Erlenmeyer flask. The culture was shaken at RT, 180 rpm for 72 h. Cultures were harvested in the late log phase of growth by centrifugation (SLA-3000 rotor, ThermoScientific) at 6000 rpm for 30 min at 4 °C. Culture supernatants were decanted into a clean, acid-washed Erlenmeyer flask containing 100 g of Amberlite XAD-4 polystyrene resin, which was shaken at 120 rpm for 4 h at 4 °C. The resin was filtered from the supernatant, rinsed with 100 mL of 90/10% ddH_2_O/MeOH, and then eluted with 250 mL of 95 : 5% MeOH/ddH_2_O. The eluent was concentrated under reduced pressure to a volume of 30 mL and stored at 4 °C prior to analysis. Frederiksenibactin and the related monocatechol and dicatechol compounds were purified by semi-preparative RP-HPLC on a YMC-Actus 20 × 250 mm C18 ODS-AQ column using a linear gradient of 15% MeOH in ddH_2_O (+0.1% trifluoroacetic acid) to 40% MeOH in ddH_2_O (+0.1% trifluoroacetic acid) over 25 min.

## Data availability

The draft genome sequence of *Dickeya chrysanthemi* EC16 was deposited at NCBI under the BioProject ID PRJNA690813.

## Author contributions

P. R. S. synthesized the compounds, isolated the FSB siderophore and carried out its structural and CD characterization; Z. L. R. carried out the bioinformatics analysis and biosynthetic gene cluster prediction; T. C. J. carried out the computational investigations. A. B. directed the project. All authors were involved in writing the manuscript and all authors provided feedback on the manuscript.

## Conflicts of interest

There are no conflicts to declare.

## Supplementary Material

SC-012-D1SC03541J-s001
