# Supplementary material for: Genomics-driven discovery of chiral triscatechol siderophores with enantiomeric Fe(iii) coordination
Source: Chem Sci. 2021 Aug 25;12(37):12485–93. doi: 10.1039/d1sc03541j (PMC8480324; doi:10.1039/d1sc03541j)
Supplement: SC-012-D1SC03541J-s001 [file SC-012-D1SC03541J-s001.pdf]

Electronic Supplementary Material (ESI) for Chemical Science.
This journal is © The Royal Society of Chemistry 2021

Electronic Supporting Information for

Genomics-driven discovery of chiral triscatechol siderophores with enantiomeric Fe(III) coordination

Parker R. Stow,[a] Zachary L. Reitz,[a] Timothy C. Johnstone,[b] Alison Butler[a,*]

[a]Department of Chemistry and Biochemistry, University of California, Santa Barbara, California 93106-9510, [b]Department of Chemistry, University of California, Santa Cruz, California 95064

Figure S1. Structures of select triscatecholate siderophores
Scheme S1. Synthetic scheme for cyclic (DHB$^{L/D}$Lys$^{L/D}$Ser)$_3$ diastereomers
Figure S2. ^{1}H NMR of cyclic (DHB^{L}Lys^{L}Ser)$_3$
Figure S3. ^{13}C NMR of cyclic (DHB^{L}Lys^{L}Ser)$_3$
Figure S4. ^{1}H-^{13}C HMBC NMR of cyclic (DHB^{L}Lys^{L}Ser)$_3$
Table S1. NMR data for cyclic (DHB^{L}Lys^{L}Ser)$_3$
Table S2. NMR data for cyclic (DHB^{D}Lys^{L}Ser)$_3$
Figure S5. HPLC chromatograms of FDAA-derivatized (DHB^{L}Lys^{L}Ser)$_3$ and (DHB^{L}Lys^{D}Ser)$_3$ hydrolysate
Figure S6. HPLC chromatograms of FDAA-derivatized (DHB^{D}Lys^{L}Ser)$_3$ and (DHB^{D}Lys^{D}Ser)$_3$ hydrolysate
Table S3. Sequencing and assembly statistics for *Dickeya chrysanthemi* EC16
Table S4. Annotation of cyclic trichrysobactin gene cluster in *Dickeya chrysanthemi* EC16
Table S5. Annotation of frederiksenibactin gene cluster in *Yersinia frederiksenii* ATCC 33641
Table S6. Complete genomes with a putative (DHB^{L}Lys^{L}Ser)$_3$ siderophore
Figure S7. HPLC of the MeOH XAD-4 extract from the supernatant of a *Y. frederiksenii* ATCC 33641 culture and ESI-MS overlays of peaks 1 and 2
Figure S8. HR-ESI-MS (a) and ESI-MSMS (b) of frederiksenibactin
Figure S9. HPLC chromatograms of FDAA-derivatized frederiksenibactin hydrolysate
Figure S10. ^{1}H NMR of frederiksenibactin (full spectrum)
Figure S11. ^{1}H NMR of frederiksenibactin (expansion of 1.2 ppm – 5.2 ppm region)
Figure S12. ^{1}H NMR of frederiksenibactin (expansion of 6.4 ppm – 12 ppm region)
Figure S13. ^{13}C NMR of frederiksenibactin
Figure S14. ^{1}H-^{13}C HMBC of frederiksenibactin
Table S7. NMR data for frederiksenibactin
Table S8. Optimized Cartesian coordinates (Å) of Δ-Fe(III)-[(DHB^{L}Lys^{L}Ser)$_3$]
Table S9. Optimized Cartesian coordinates (Å) of Δ-Fe(III)-[(DHB^{D}Lys^{D}Ser)$_3$]
Table S10. Optimized Cartesian coordinates (Å) of Δ-Fe(III)-[(DHB^{L}Lys^{D}Ser)$_3$]
Table S11. Optimized Cartesian coordinates (Å) of Δ-Fe(III)-[(DHB^{D}Lys^{L}Ser)$_3$]

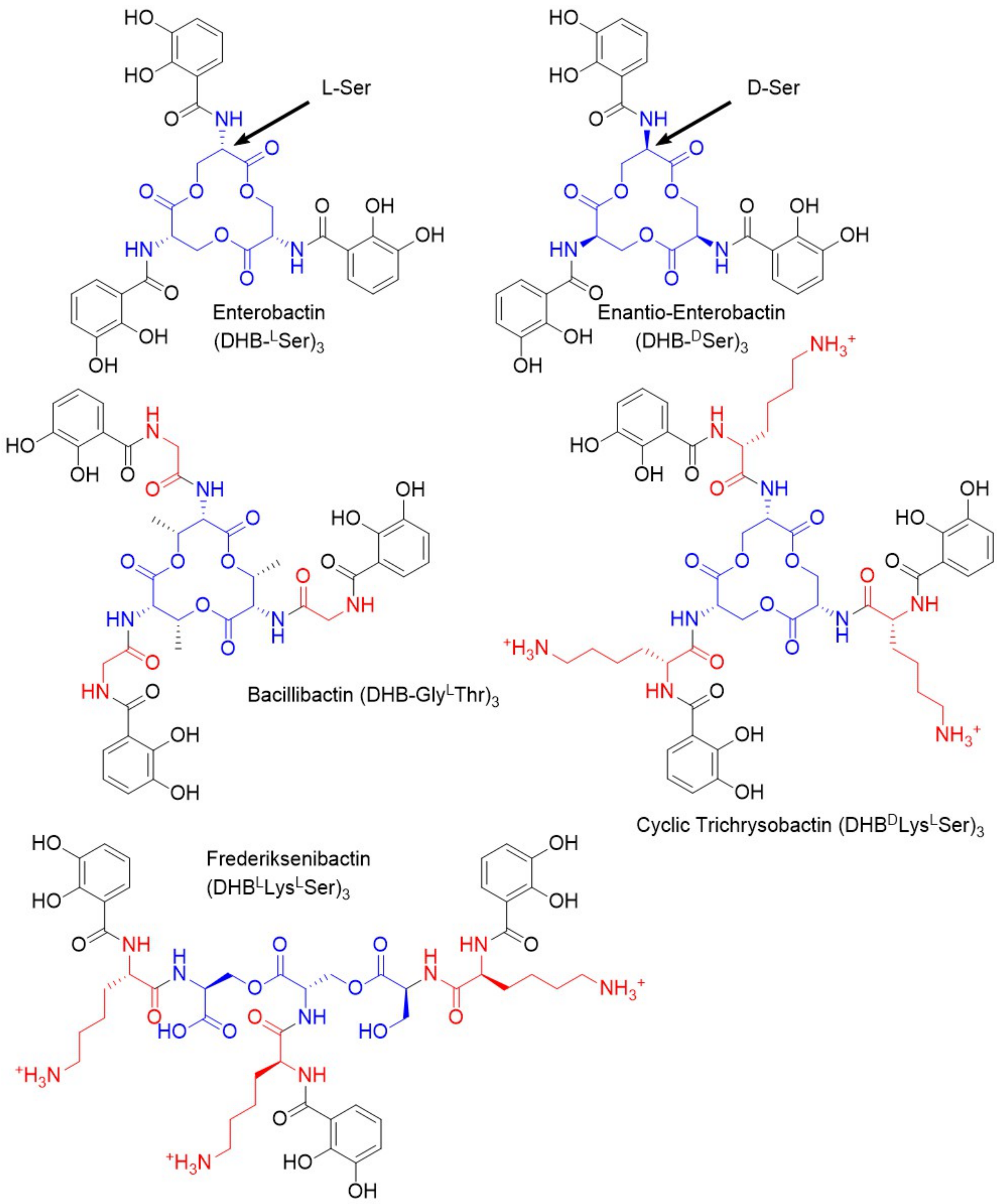

Figure S1. Structures of select triscatechol siderophores

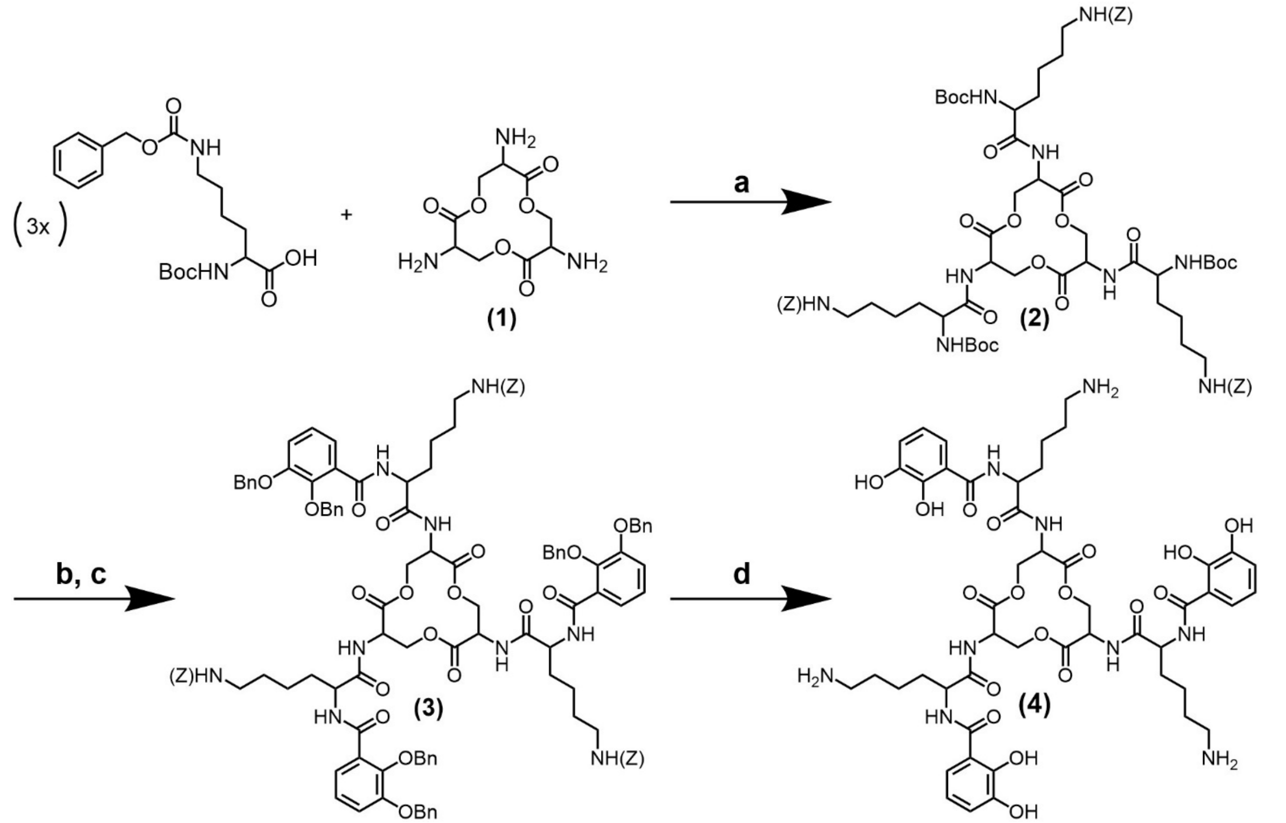

Scheme S1. Synthetic scheme for cyclic (DHB$^{L/D}$Lys$^{L/D}$Ser)$_3$ diastereomers.

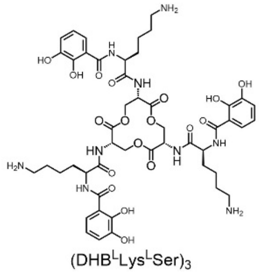

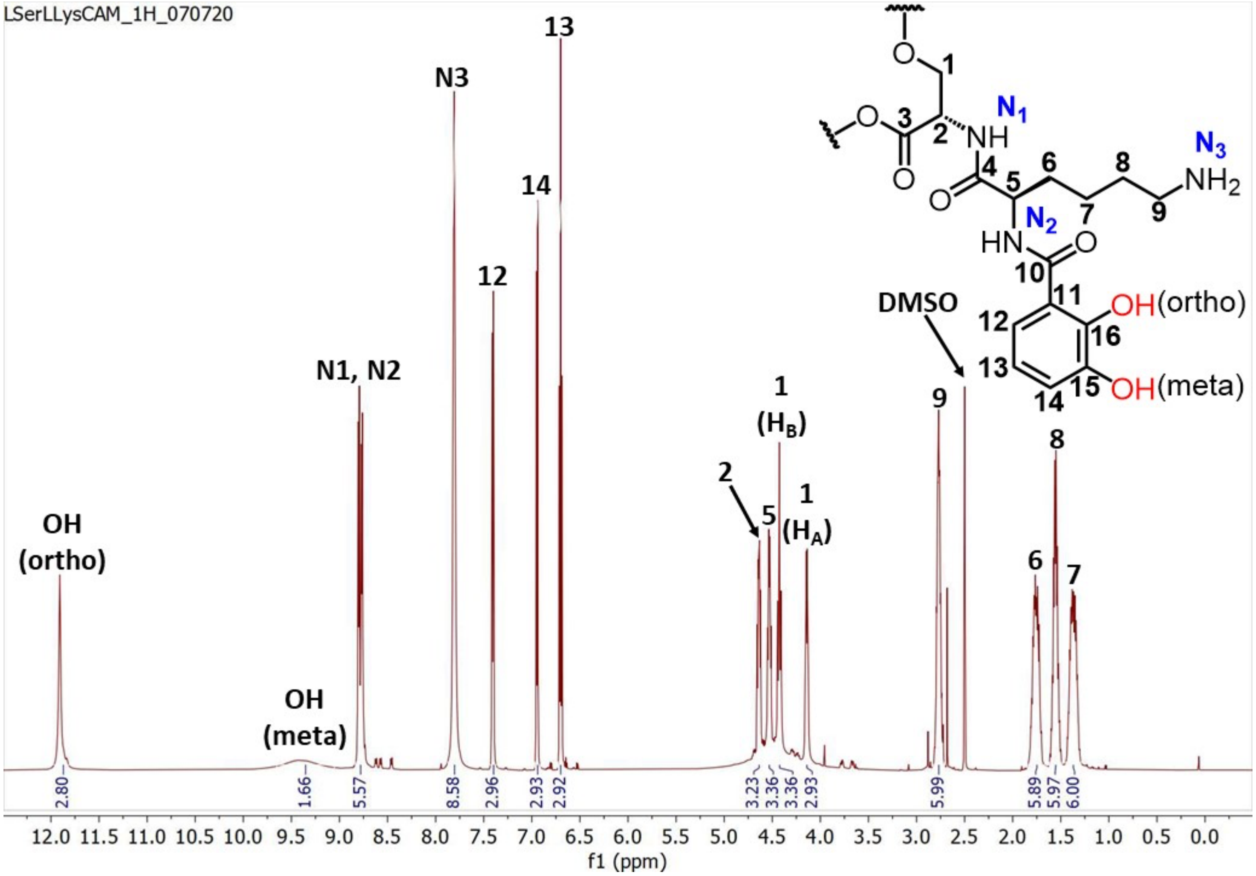

Figure S2: ^{1}H NMR of cyclic (DHB^{L}Lys^{L}Ser)$_3$ in $(CD_3)_2SO$.

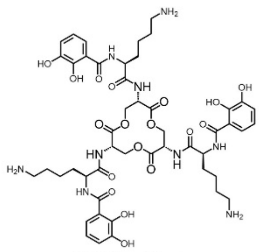

(DHB^{L}Lys^{L}Ser)$_3$

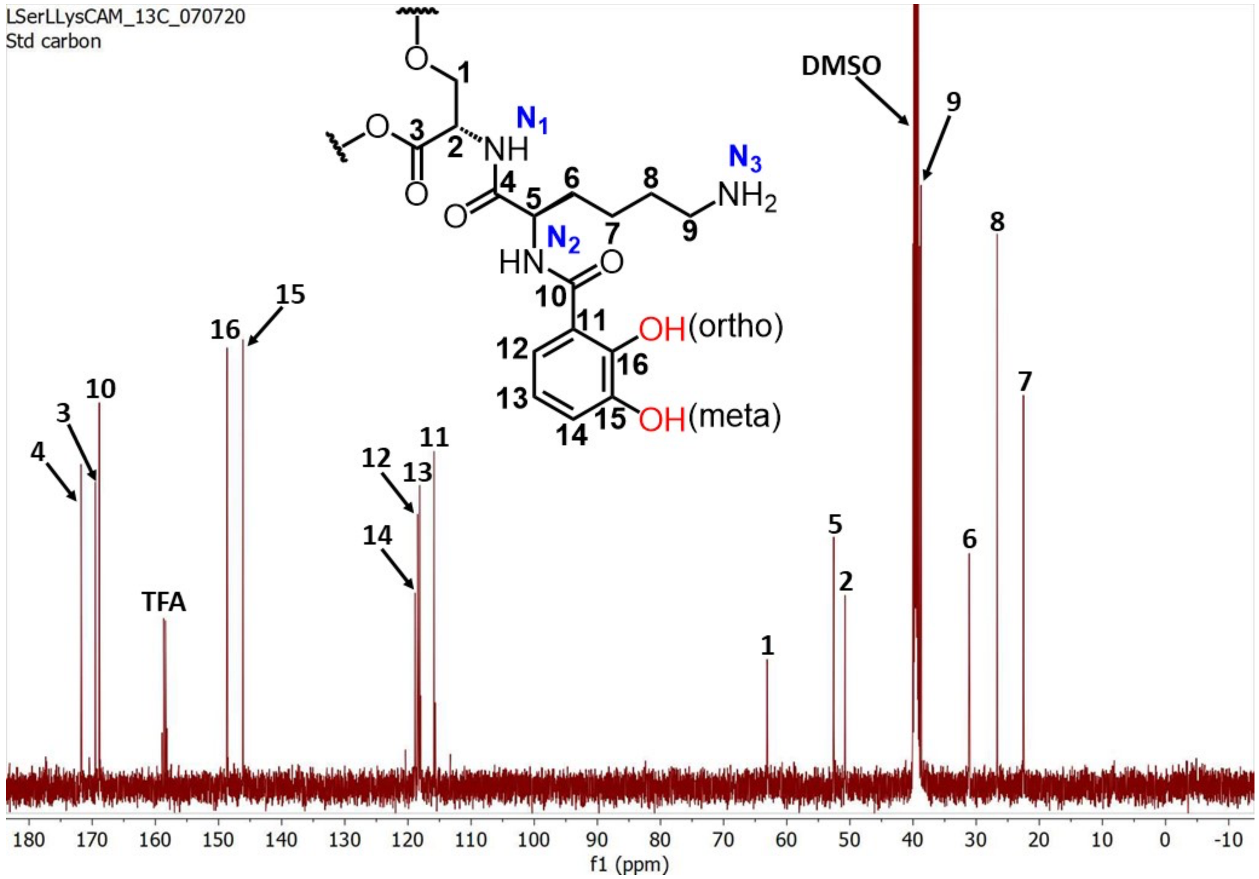

Figure S3. ^{13}C NMR of cyclic (DHB^{L}Lys^{L}Ser)$_3$ in $(CD_3)_2SO$.

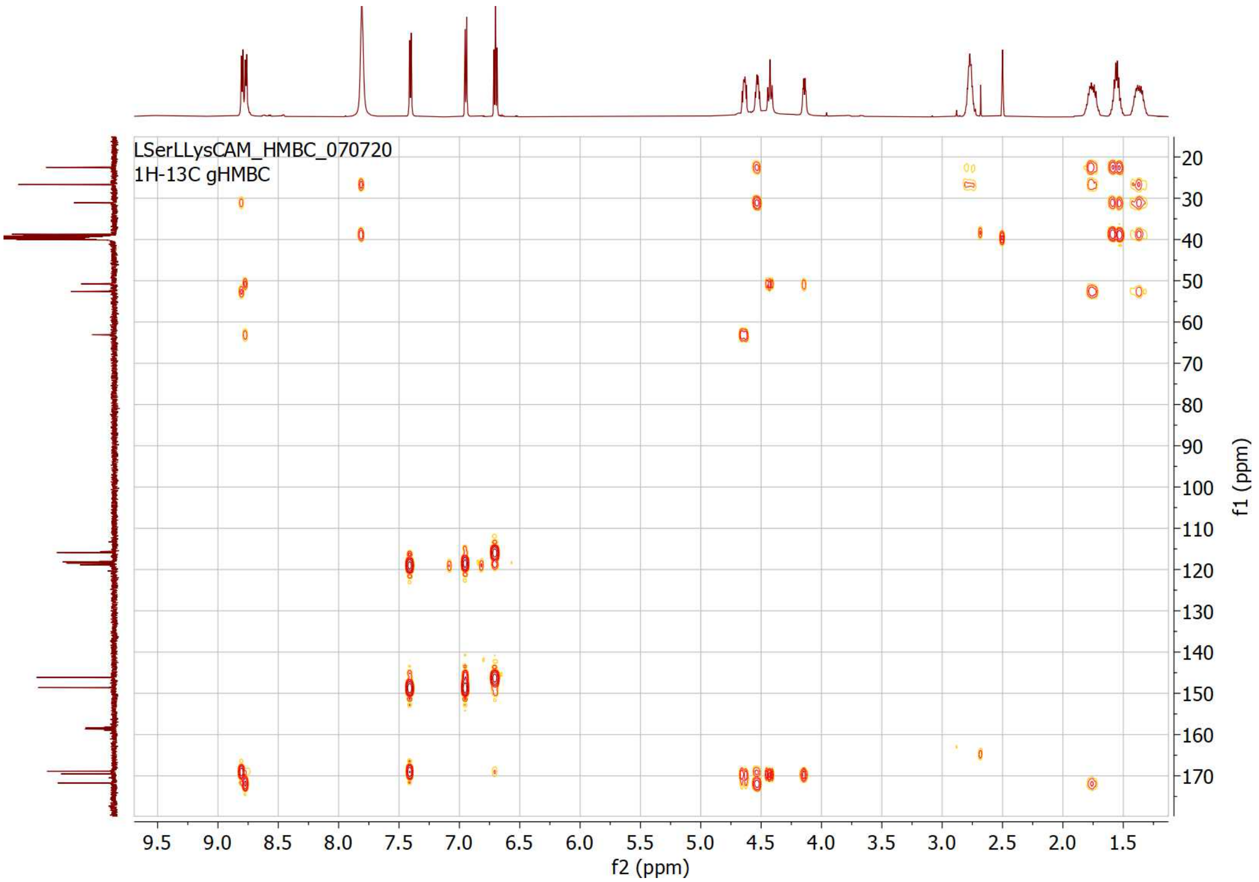

Figure S4. ^{1}H-^{13}C HMBC NMR of cyclic (DHB^{L}Lys^{L}Ser)$_3$ in $(CD_3)_2SO$.

Table S1. NMR data for cyclic (DHB^{L}Lys^{L}Ser)$_3$ in $(CD_3)_2SO$. HMBC correlations are from proton(s) stated to the indicated carbon.

(DHB^{L}Lys^{L}Ser)$_3$			
Position	δ_C, type	δ_H(*J* in Hz)	HMBC
1 (H_A)	63.1, CH_2	4.43, t (10.6)	2, 3
1 (H_B)	63.1, CH_2	4.14, dd (10.8, 6.2)	2, 3
2	50.8, CH	4.64, ddd (10.1, 7.2, 4.7)	1, 3
3	169.5, C		
4	171.6, C		
5	52.6, CH	4.53, td (8.5, 4.8)	4, 6, 7, 10
6	31.1, CH_2	1.75, m	4, 5, 7, 8
7	22.5, CH_2	1.37, m	5, 6, 8, 9
8	26.7, CH_2	1.55, m	6, 7, 9
9	38.7, CH_2	2.77, m	8
10	168.9, C		
11	115.9, C		
12	118.4, CH	7.40, d (8.2)	10, 11, 13, 16
13	118.2, CH	6.70, t (7.9)	10, 11, 12, 15
14	118.9, CH	6.95, d (7.8)	12, 15, 16
15	146.1, C		
16	148.6, C		
N1		8.77, d (7.2)	1, 2, 4
N2		8.80, d (7.6)	5, 6, 10
N3		7.81, s	8, 9
OH (meta)		9.40, s	-
OH (ortho)		11.91, s	-

Table S2. NMR data for cyclic (DHB^{D}Lys^{L}Ser)$_3$ in $(CD_3)_2SO$. HMBC correlations are from proton(s) stated to the indicated carbon. Indicated positions correspond to the analogous positions in (DHB^{L}Lys^{L}Ser)$_3$ (Table S1).

(DHB^{D}Lys^{L}Ser)$_3$			
Position	δ_C, type	δ_H(J in Hz)	HMBC
1 (H_A)	63.1, CH_2	4.44, t (10.5)	2, 3
1 (H_B)	63.1, CH_2	4.12, dd (10.8, 4.7)	2, 3
2	50.7, CH	4.65, ddd (10.2, 7.5, 4.7)	1, 3
3	169.5, C		
4	171.7, C		
5	52.5, CH	4.55, td (8.4, 5.2)	4, 6, 7, 10
6	31.3, CH_2	1.75, m	4, 5, 7, 8
7	22.5, CH_2	1.34, m	5, 6, 8, 9
8	26.7, CH_2	1.54, m	6, 7, 9
9	38.7, CH_2	2.77, s	-
10	168.7, C		
11	115.9, C		
12	118.5, CH	7.41, d (8.2)	10, 11, 14, 16
13	118.2, CH	6.70, t (7.9)	10, 11, 14, 15
14	118.8, CH	6.94, d (7.9)	12, 16
15	146.1, C		
16	148.5, C		
N1		8.80, d (overlapping with N2)	1, 2, 4
N2		8.80, d (overlapping with N1)	5, 6, 10
N3		7.78, s	-
OH (meta)		9.44, s	-
OH (ortho)		11.86, s	11, 15

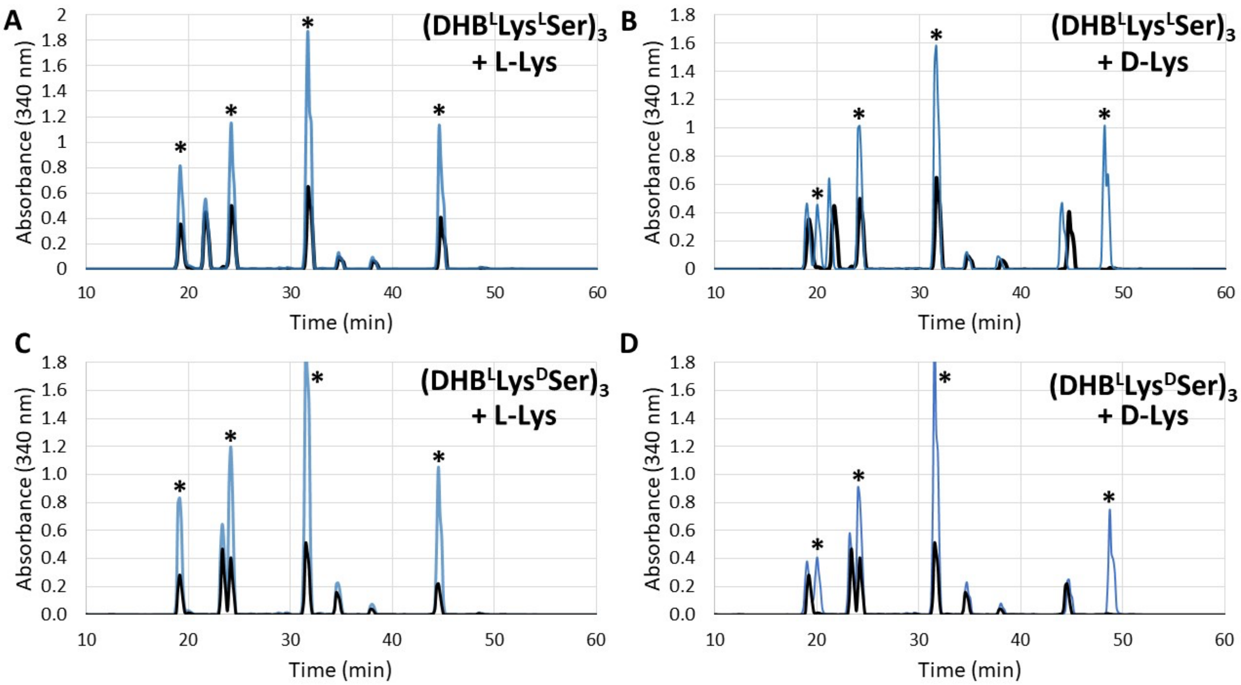

Figure S5. HPLC chromatograms of (DHB^{L}Lys^{L}Ser)$_3$ and (DHB^{L}Lys^{D}Ser)$_3$ hydrolysate derivatized with FDAA (light blue trace) overlayed with FDAA-derivatized amino acid standards (black trace). Derivatized hydrolysis products were separated by HPLC on a YMC 4.6 x 250 mm C18-A column using a gradient from 10% CH_3CN in TEAP buffer (50 mM, pH 3.00) to 40% CH_3CN in TEAP over 60 min. Asterisks indicate the peaks that increase upon addition of the designated amino acid standard.

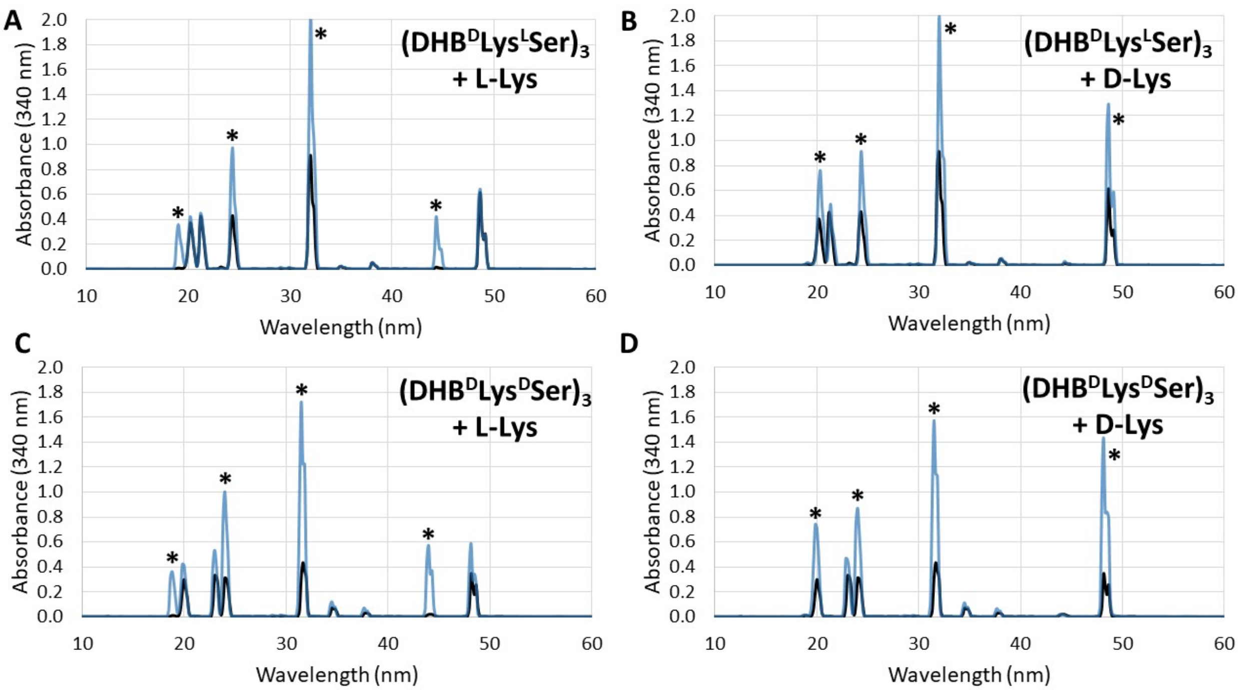

Figure S6. HPLC chromatograms of $(DHB^{D}Lys^{L}Ser)_3$ and $(DHB^{D}Lys^{D}Ser)_3$ hydrolysate derivatized with FDAA (light blue trace) overlayed with FDAA-derivatized amino acid standards (black trace). Derivatized hydrolysis products were separated by HPLC on a YMC 4.6 x 250 mm C18-A column using a gradient from 10% CH_3CN in TEAP buffer (50 mM, pH 3.00) to 40% CH_3CN in TEAP over 60 min. Asterisks indicate the peaks that increase upon addition of the designated amino acid standard.

Table S3. Sequencing and assembly statistics for *Dickeya chrysanthemi* EC16 (=ATCC 11662)

Statistic	Data for *Dickeya chrysanthemi* EC16
Total filtered read length (bp)	865,361,754
Assembly size (bp)	4,685,942
Coverage (×)	185
No. of scaffolds	70
No. of contigs	76
Scaffold N_{50} (bp)	190,138
Contig N_{50} (bp)	186,974
G+C content (%)	54.36
No. of identified genes (total)	4,205
No. of identified CDSs (total)	4,136
No. of rRNAs (5S, 16S, 23S)	1, 2, 1
No. of predicted tRNAs	58
No. of predicted ncRNAs	7
Genome accession no.	JAFCAF000000000
SRA accession no.	SRR13439815

Table S4. Annotation of cyclic trichrysobactin gene cluster in *Dickeya chrysanthemi* EC16, including predicted protein functions based on sequence analysis using Pfam and BLAST. The sequence similarity of each gene to its corresponding homolog from the chrysobactin locus (*Dickeya dadantii* 3937) is indicated.

			**Homologs in *D. dadantii* 3937**			
**Name**		**Locus tag**	**%ID**	**%SIM**	**Locus tag**	**Accession**
cbsA	2,3-dihydro-2,3-DHB dehydrogenase	JJO56_07665	90	92	DDA3937_RS14720	WP_013318793
cbsB	Isochorismatase / Aryl carrier protein	JJO56_07670	95	96	DDA3937_RS14715	WP_013318792
cbsE	2,3-DHB–AMP ligase	JJO56_07675	92	95	DDA3937_RS14710	WP_013318791
cbsC	Isochorismate synthase	JJO56_07680	92	94	DDA3937_RS14705	WP_013318790
fctA	TonB-dependent receptor	JJO56_07685	81	88	DDA3937_RS14700	WP_013318789
cbsH	Esterase	JJO56_07690	88	92	DDA3937_RS14695	WP_033111969
cbsI	MbtH-like protein	JJO56_07695	86	90	DDA3937_RS14690	WP_013318786
cbsF	NRPS	JJO56_07700	91	94	DDA3937_RS14685	WP_033112377
fctC	ABC ATP-binding component	JJO56_07705	96	96	DDA3937_RS14675	WP_033111968
fctG	ABC permease	JJO56_07710	90	94	DDA3937_RS14670	WP_033112376
fctD	ABC permease	JJO56_07715	92	95	DDA3937_RS14665	WP_013318781
cbsS	MFS transporter	JJO56_07720	96	97	DDA3937_RS14660	WP_013318779
fctB	ABC periplasmic binding protein	JJO56_07725	84	90	DDA3937_RS14655	WP_033111967

Table S5. Annotation of frederiksenibactin gene cluster of *Yersinia frederiksenii* ATCC 33641. The predicted protein functions based on sequence analysis using Pfam and BLAST are included. The sequence similarity of each gene to its corresponding homolog in the cyclic trichrysobactin locus (*D. chrysanthemi* EC16, Table S2) is indicated.

				Homologs in *D. chrysanthemi* EC16		
**Name**	**Putative function**	**Locus tag**	**Accession**	**Name**	**%ID**	**%SIM**
ffuA	TonB-dependent receptor	DJ58_RS13925	WP_004709694	*fctA*	54	71
freH	Esterase	DJ58_RS13920	WP_080544642	*cbsH*	49	62
freI	MbtH-like protein	DJ58_RS13915	WP_032911217	*cbsI*	63	76
freF	NRPS	DJ58_RS13910	WP_050504533	*cbsF*	58	71
ffuC	ABC ATP-binding component	DJ58_RS13905	WP_080544678	*fctC*	71	84
ffuG	ABC permease	DJ58_RS13900	WP_032911802	*fctG*	63	77
ffuD	ABC permease	DJ58_RS13895	WP_004711942	*fctD*	59	77
freS	MFS transporter	DJ58_RS13890	WP_004711941	*cbsS*	64	77
ffuB	ABC periplasmic binding protein	DJ58_RS13885	WP_004711939	*fctB*	54	72
freC	Isochorismate synthase	DJ58_RS13880	WP_004711937	*cbsC*	47	62
freE	2,3-DHB–AMP ligase	DJ58_RS13875	WP_032911801	*cbsE*	64	74
freB	Isochorismatase / Aryl carrier protein	DJ58_RS13870	WP_004711933	*cbsB*	58	74
freA	2,3-dihydro-2,3-DHB dehydrogenase	DJ58_RS13865	WP_032911814	*cbsA*	65	77

Table S6. Complete genomes with a putative (DHB^{L}Lys^{L}Ser)$_3$ siderophore.

Strain	RefSeq Accession
Yersinia enterocolitica LC20	GCF_000597945.1
Y. kristensenii Y231	GCF_000834865.1
Y. frederiksenii Y225	GCF_000834215.1
Y. frederiksenii FDAARGOS_417	GCF_002591095.1
Y. frederiksenii FDAARGOS_418	GCF_002591195.1
Y. massiliensis GTA	GCF_003048255.1
Y. hibernica CFS1934	GCF_004124235.1

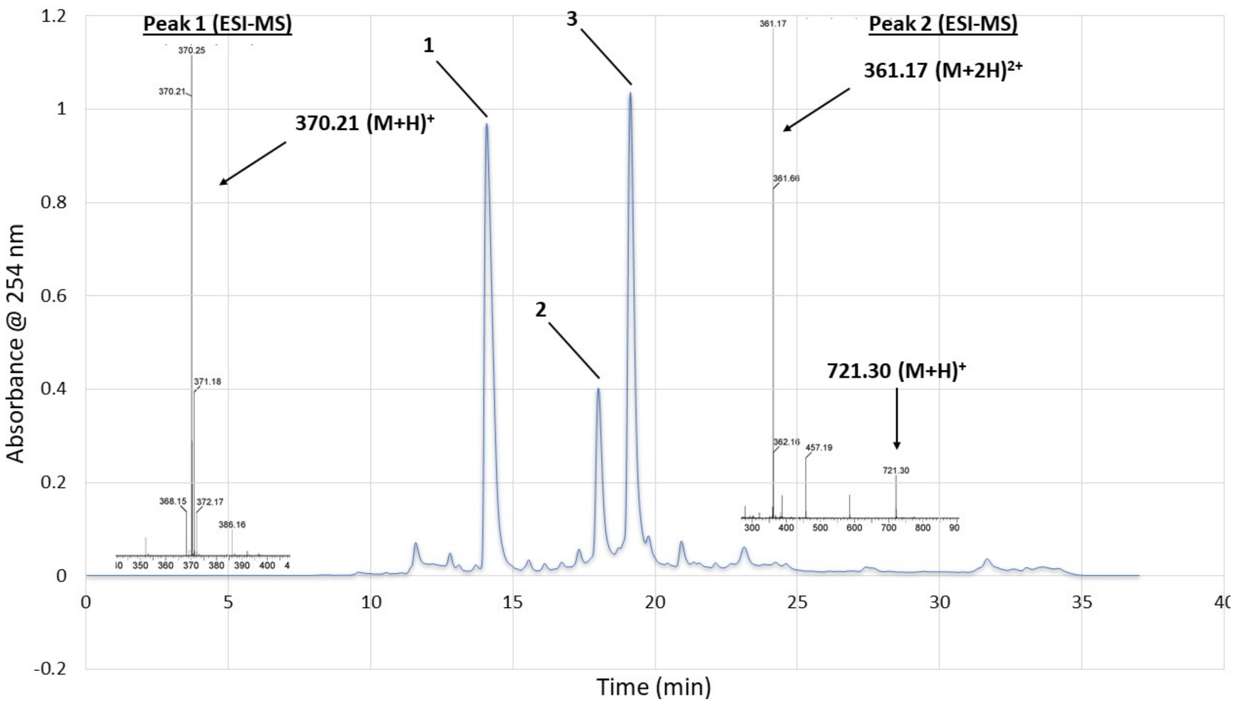

Figure S7. HPLC of the MeOH XAD-4 extract from the supernatant of a *Y. frederiksenii* ATCC 33641 culture. ESI-MS of isolated peaks **1** and **2** corresponding to the related monocatechol and dicatechol fragments of frederiksenibactin, respectively. MS data for peak **3** corresponding to frederiksenibactin shown in Figure S8.

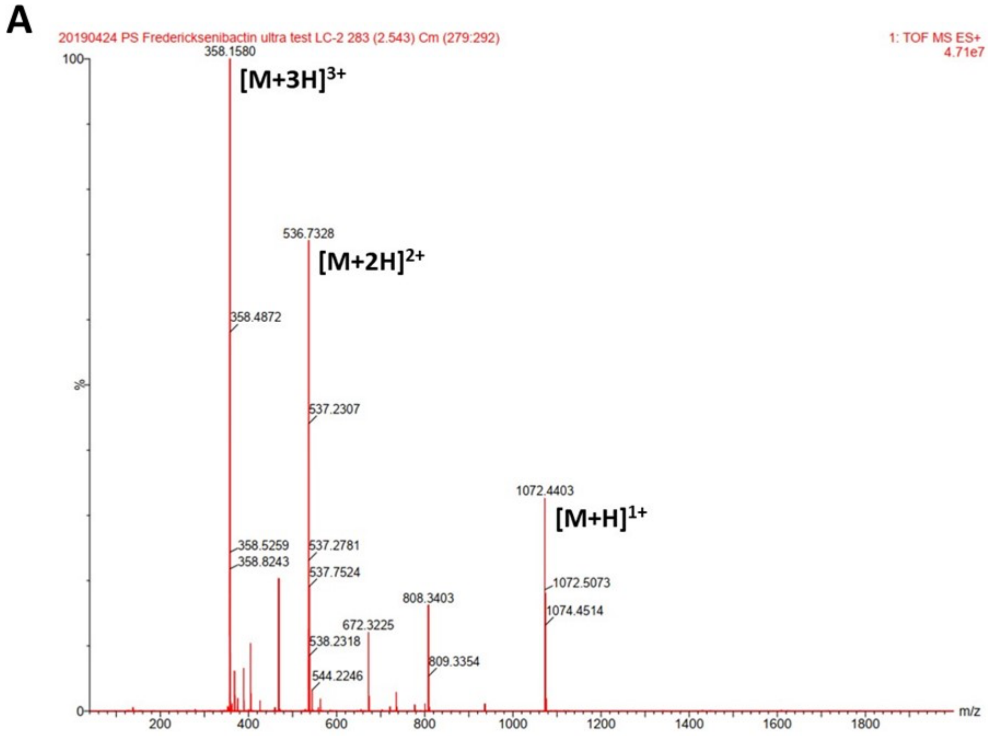

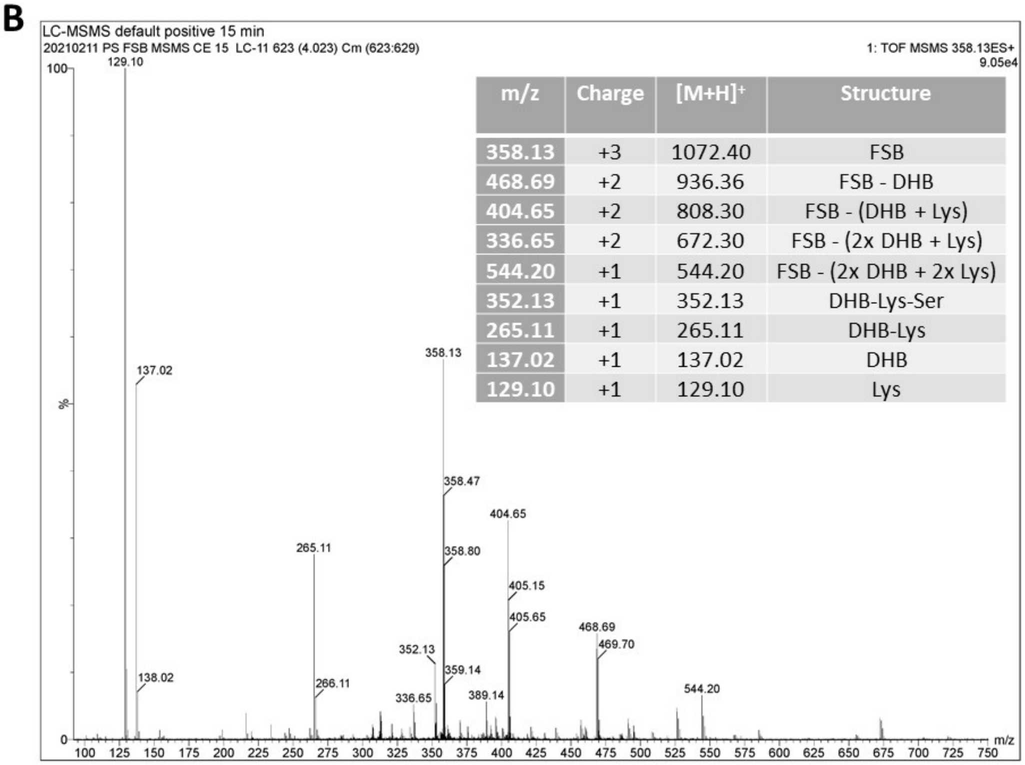

m/z	Charge	$[M+H]^+$	Structure
358.13	+3	1072.40	FSB
468.69	+2	936.36	FSB - DHB
404.65	+2	808.30	FSB - (DHB + Lys)
336.65	+2	672.30	FSB - (2x DHB + Lys)
544.20	+1	544.20	FSB - (2x DHB + 2x Lys)
352.13	+1	352.13	DHB-Lys-Ser
265.11	+1	265.11	DHB-Lys
137.02	+1	137.02	DHB
129.10	+1	129.10	Lys

Figure S8. (a) HR-ESI-MS of frederiksenibactin. Observed *m/z* 1072.4403 $[M+H]^{1+}$. Calculated exact mass for frederiksenibactin $[M+H]^{1+}$ is 1072.4475 ($C_{48}H_{66}N_9O_{19}$). (b) ESI-MSMS of frederiksenibactin.

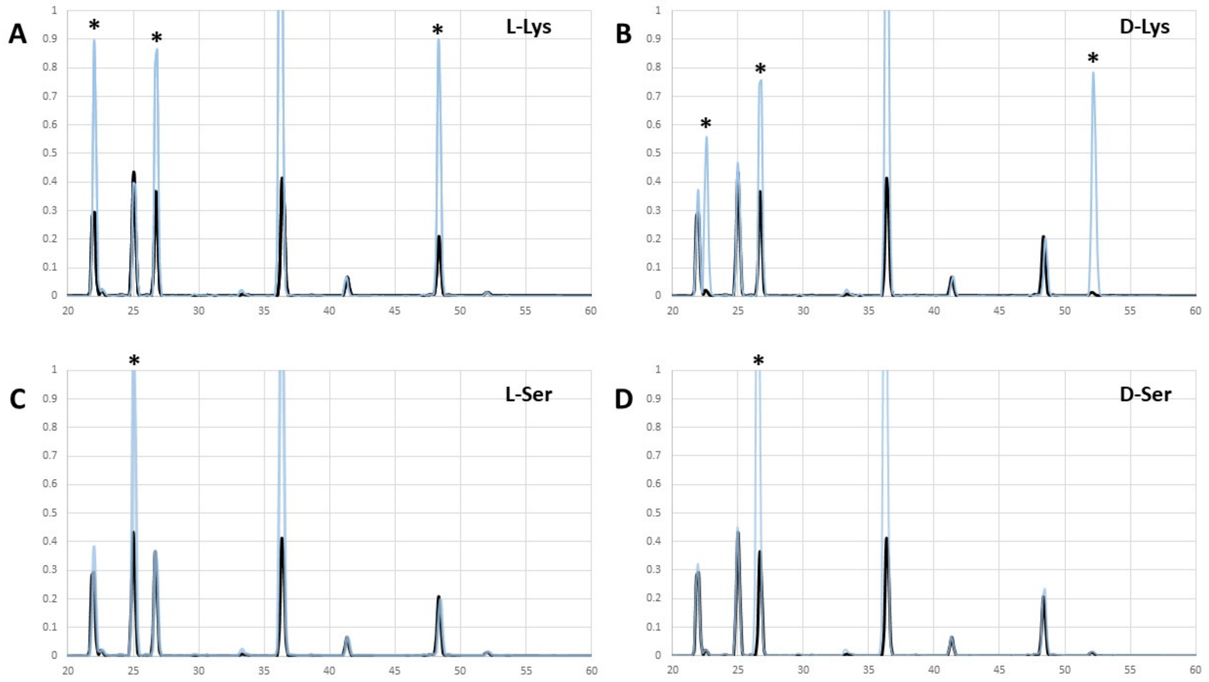

Figure S9. HPLC chromatograms of frederiksenibactin hydrolysate derivatized with FDAA (light blue trace) overlayed with FDAA-derivatized amino acid standards (black traces). Derivatized hydrolysis products were separated by HPLC on a YMC 4.6 x 250 mm C18-AQ column using a gradient from 10% CH_3CN in ddH_2O (0.05% trifluoroacetic acid) to 40% CH_3CN in ddH_2O (0.05% trifluoroacetic acid) over 60 min. Asterisks indicate the peaks that increase upon addition of the designated amino acid standard.

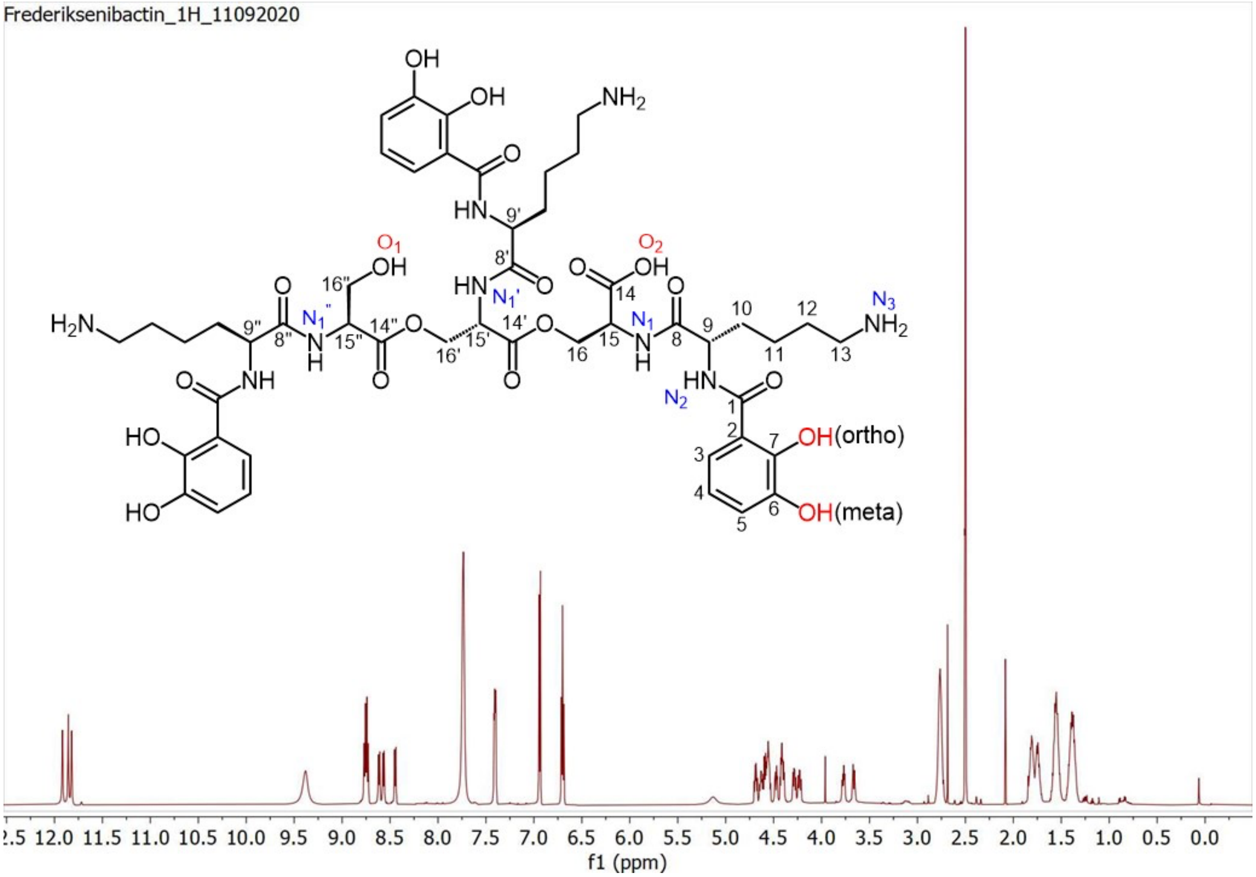

Figure S10. ^{1}H NMR of frederiksenibactin, FSB, in $(CD_3)_2SO$.

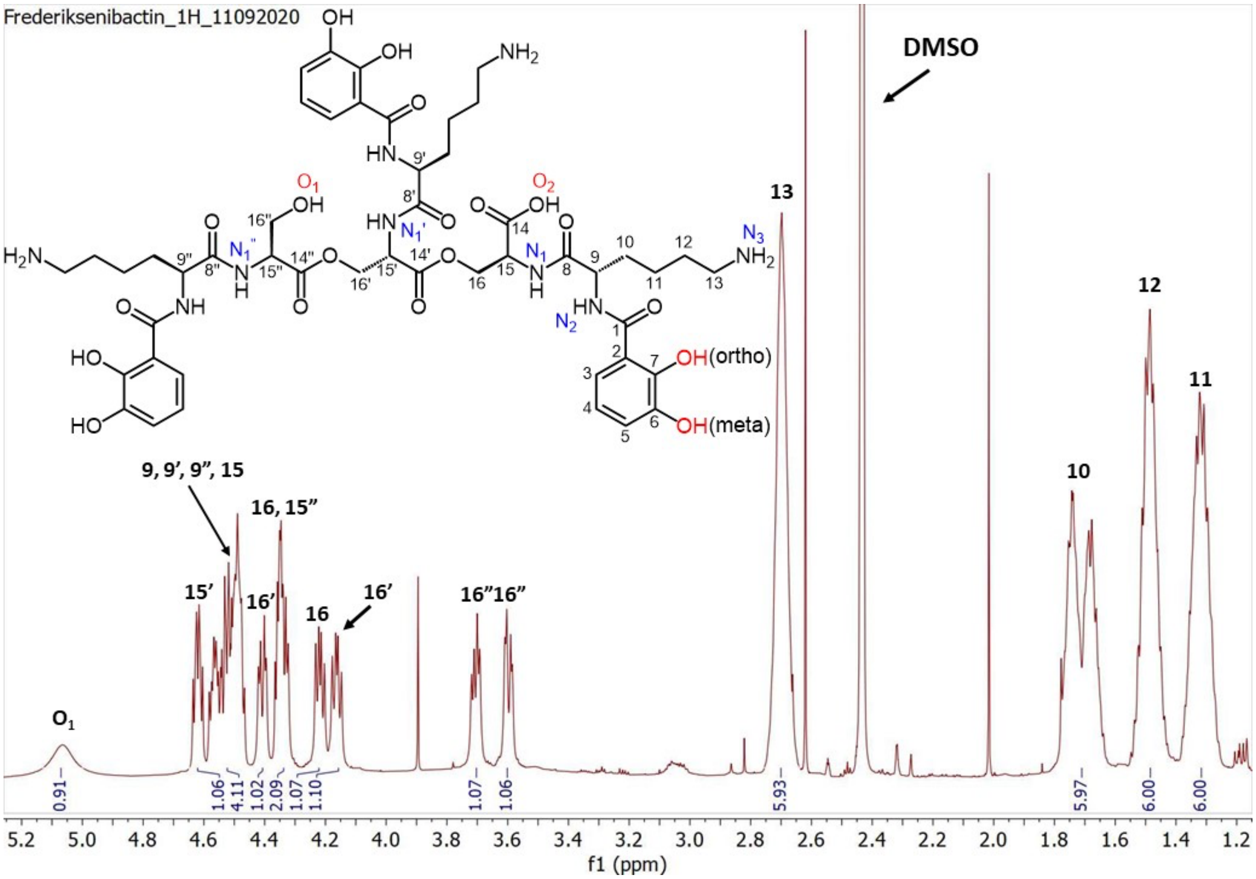

Figure S11. ^{1}H NMR of frederiksenibactin (expansion of 1.2 ppm – 5.2 ppm region) in $(CD_3)_2SO$.

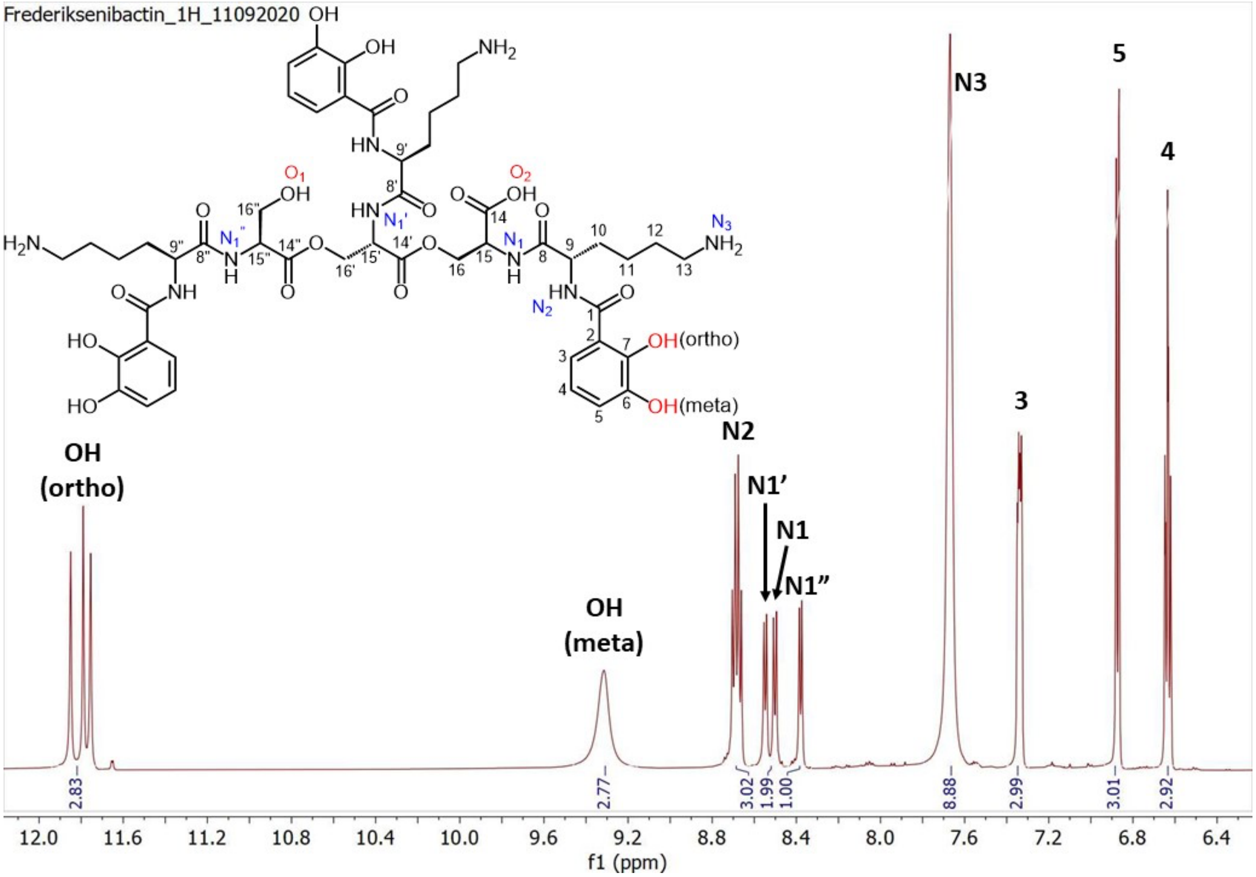

Figure S12. ^{1}H NMR of frederiksenibactin (expansion of 6.4 ppm – 12.0 ppm region) in $(CD_3)_2SO$.

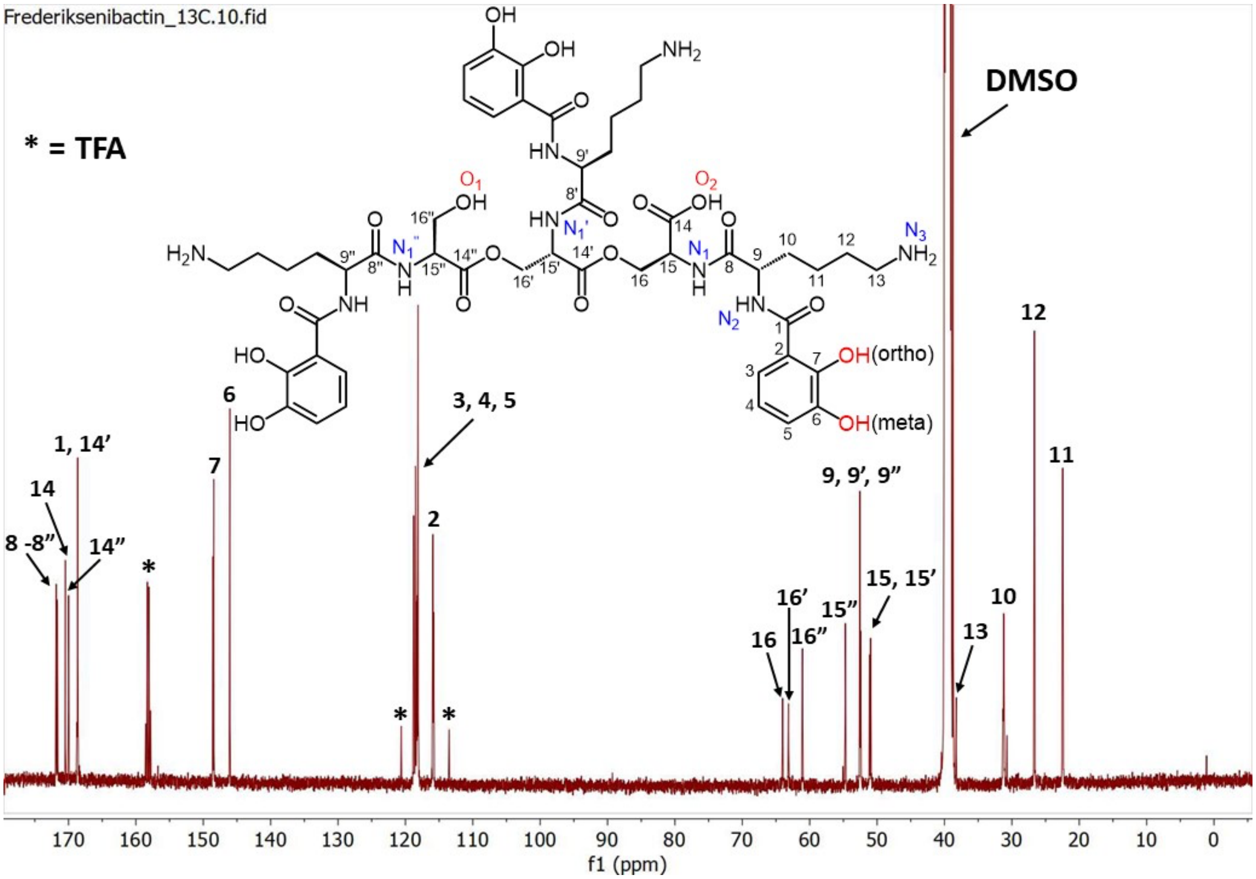

Figure S13. ^{13}C NMR of frederiksenibactin in $(CD_3)_2SO$. TFA observed in all ^{13}C NMR spectra following HPLC purification, where it is used as an ion-pairing agent.

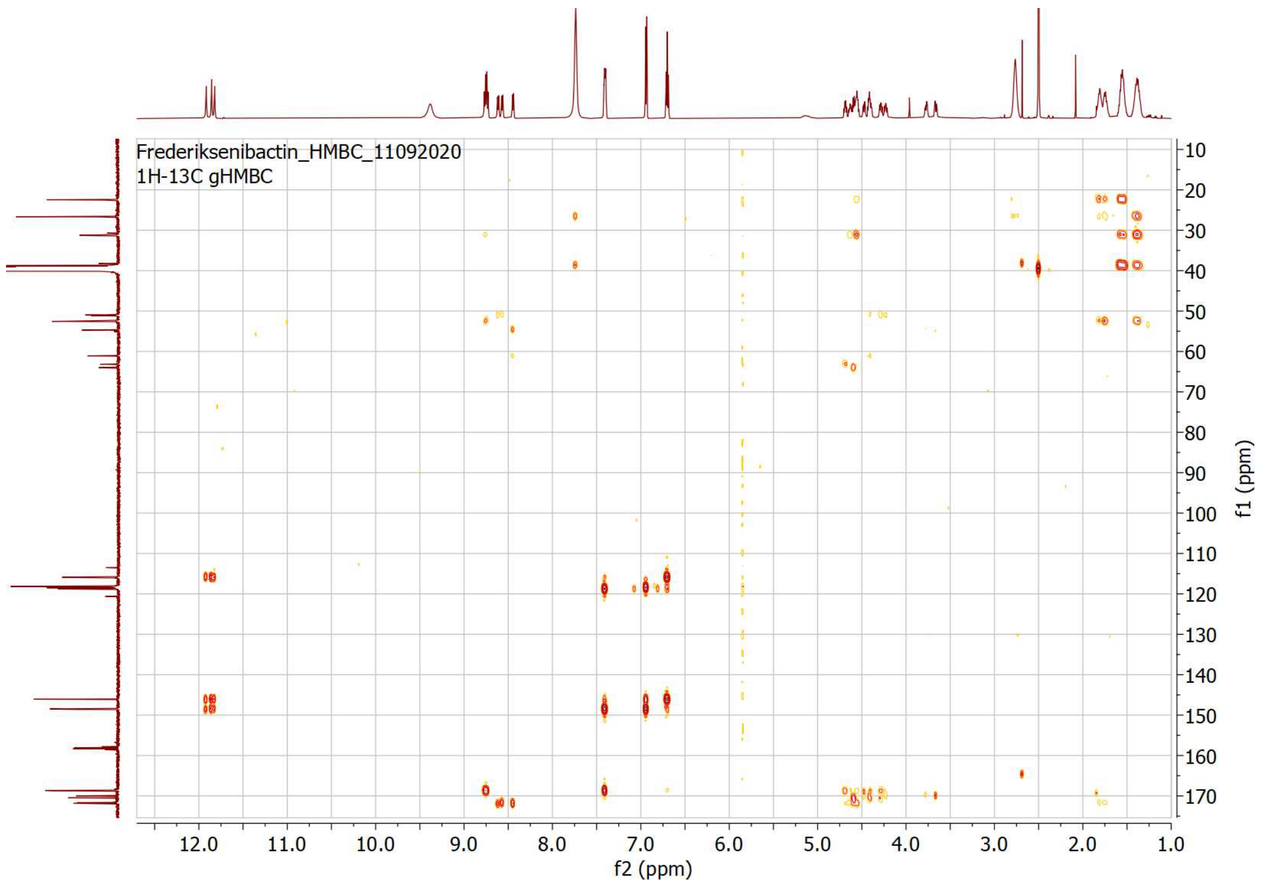

Figure S14. ^1H-^{13}C HMBC of frederiksenibactin (FSB) in $(CD_3)_2SO$.

Table S7. NMR data for frederiksenibactin in $(CD_3)_2SO$. HMBC correlations are from proton(s) stated to the indicated carbon.

Frederiksenibactin **(1)**			
Position	δ_C, type	δ_H(J in Hz)	HMBC
1	168.7, C		
2	115.9, C		
3	118.5, CH	7.40, m	1, 2, 5, 6, 7
4	118.1, CH	6.70, td (7.9, 2.2)	3, 5, 6, 7
5	118.8, CH	6.94, d (7.8)	3, 6, 7
6	146.1, C		
7	148.5, C		
8	171.7, C		
8'	171.9, C		
8"	171.8, C		
9	52.5, CH	4.65, m	8, 10, 11
9' / 9"	52.6, CH	4.50 - 4.60, m	8', 8", 10, 11
10 (H_A)	31.2, CH_2	1.75, m	9, 11, 12
10 (H_B)	31.2, CH_2	1.80, m	9, 11, 12
11	22.4, CH_2	1.39, m	9, 10, 12, 13
12	26.7, CH_2	1.55, m	10, 11, 13
13	38.7, CH_2	2.77, m	
14	170.5, C		
15	51.0, CH	4.59, m	14, 16
16 (H_A)	64.0, CH_2	4.28, dd(11.1, 6.4)	14, 15, 17
16 (H_B)	64.0, CH_2	4.40, m	15, 17
14'	168.7, C		
15'	51.1, CH	4.69, td (7.2, 4.6)	
16' (H_A)	63.2, CH_2	4.23, dd (11.4, 7.0)	17, 18, 20
16' (H_B)	63.2, CH_2	4.47, dd (11.4, 4.3)	17, 20
14"	170.0, C		
15"	54.7, CH	4.41, m	20, 22
16" (H_A)	61.1, CH	3.66, dd (11.4, 4.0)	20
16" (H_B)	61.1, CH	3.77, dd (11.3, 5.3)	20
N1		8.57, d (7.7)	8, 15
N1'		8.61, d (7.9)	8', 18
N1"		8.45, d (8.4)	8", 21
N2		8.75, m	1, 9-9"
N3		7.73, br s	12, 13
O1		13.13, br s	
O2		5.13, br s	
OH (meta)		9.38, br s	
OH (ortho)		11.82 - 11.92, s	2, 6, 7

Table S8. Optimized Cartesian coordinates (Å) of Δ-Fe(III)-[(DHB^{L}Lys^{L}Ser)$_3$].

O	-1.732100	0.793600	5.333300
O	-0.385900	-2.339000	5.168900
O	1.583300	0.399500	5.420400
C	-1.791800	0.807200	4.132000
C	-0.223700	-2.275600	3.978800
C	1.629100	0.607700	4.236700
C	-2.437800	-1.527400	3.625600
C	2.166200	-1.645600	3.771000
C	-0.057800	2.403900	3.976700
H	-2.461700	-1.735000	4.695100
H	-3.153200	-2.164000	3.110400
H	2.245000	-1.641600	4.857900
H	3.122800	-1.919900	3.332800
H	-0.220200	2.388000	5.054000
H	-0.241600	3.403200	3.589400
O	1.861200	-0.319400	3.301800
O	-0.999800	1.527100	3.331400
O	-1.136800	-1.849700	3.099800
C	1.380700	1.996600	3.682600
H	1.987700	2.661800	4.305600
C	1.103500	-2.654100	3.352100
H	1.396900	-3.583400	3.850800
C	-2.790800	-0.063300	3.395800
H	-3.736100	0.077900	3.930100
N	1.072900	-2.938500	1.940900
N	-3.043800	0.285000	2.021900
N	1.795000	2.215500	2.321200
H	1.654700	1.501800	1.620300
H	0.527700	-2.365400	1.312800
H	-2.282600	0.534900	1.406800
C	2.476900	3.340100	2.001800
O	2.746500	4.208900	2.823200
C	2.838200	3.547800	0.539200
H	3.851700	3.955000	0.558300
N	2.854200	2.344300	-0.253400
H	2.048700	2.072000	-0.828600
C	1.685000	-4.048700	1.466600
O	2.299100	-4.828400	2.185500
C	1.666600	-4.264800	-0.038500
H	1.517300	-5.339600	-0.163700
N	0.604300	-3.580500	-0.730800
H	0.764000	-2.680000	-1.196700
C	-4.319600	0.377200	1.577300
O	-5.289900	0.154700	2.291800
C	-4.525700	0.686100	0.102200
H	-5.383100	1.362600	0.077300
N	-3.415500	1.347400	-0.534600
H	-2.718800	0.823300	-1.076400
C	3.022100	-3.886500	-0.658900
C	3.502200	-2.468000	-0.338300
H	2.913400	-4.022700	-1.738600
H	3.765800	-4.611600	-0.313900
C	4.350400	-2.450000	0.932100
H	4.074300	-2.071300	-1.186500
H	2.648900	-1.788400	-0.213700
C	4.744600	-1.054400	1.368400
H	3.813800	-2.932500	1.748600
H	5.252400	-3.055900	0.780500
H	3.915900	-0.353700	1.243000
H	5.073800	-1.018200	2.407500
C	-4.891300	-0.588600	-0.676500
C	-3.900200	-1.744700	-0.518000
H	-4.975500	-0.292300	-1.725800
H	-5.884800	-0.911000	-0.349500
C	-4.288000	-2.640400	0.656700
H	-3.861000	-2.327300	-1.447100
H	-2.881200	-1.364800	-0.365900
C	-3.273800	-3.728100	0.941700
H	-4.419700	-2.041200	1.557500
H	-5.268300	-3.093900	0.464700
H	-2.253300	-3.347100	0.859000
H	-3.399400	-4.166100	1.932300
C	1.909800	4.587700	-0.109800
C	0.414500	4.282400	0.017800
H	2.203800	4.645600	-1.161600
H	2.125000	5.561300	0.341400
C	-0.166400	4.906500	1.285200
H	-0.115000	4.653400	-0.868900
H	0.239800	3.198500	0.033800
C	-1.611200	4.527400	1.532500
H	0.424200	4.613000	2.152000
H	-0.078100	5.998600	1.231200
H	-1.786600	3.474000	1.302600
H	-1.921300	4.711500	2.561700
C	-0.523000	-4.224900	-1.100400
O	-0.829600	-5.325400	-0.589800
C	4.011500	1.719800	-0.557800
O	5.059200	1.962300	0.082500
C	-3.419700	2.680300	-0.746100
O	-4.218500	3.424400	-0.133100
C	-2.481100	3.203300	-1.750100
C	-1.494400	2.389200	-2.336500
C	-2.653400	4.527900	-2.221400
C	-0.722700	2.885800	-3.446000
C	-1.842700	5.029300	-3.218100
H	-3.475800	5.119100	-1.832300
C	-0.875400	4.212200	-3.831300
H	-1.982400	6.047600	-3.566900
H	-0.268200	4.595000	-4.647000
C	3.989700	0.782500	-1.690800
C	2.788200	0.405900	-2.319400
C	5.221100	0.339300	-2.232900
C	2.827700	-0.368000	-3.533400
C	5.245900	-0.484600	-3.338600
H	6.146400	0.710300	-1.804400
C	4.051500	-0.842200	-3.989900
H	6.196500	-0.821400	-3.740100
H	4.075700	-1.454900	-4.886800
C	-1.337300	-3.599100	-2.153200
C	-1.068200	-2.301000	-2.625500
C	-2.339900	-4.371300	-2.789600
C	-1.762200	-1.803100	-3.784900
C	-3.070800	-3.848600	-3.835900
H	-2.478500	-5.403100	-2.484100
C	-2.786300	-2.564500	-4.334800
H	-3.839700	-4.449700	-4.311000
H	-3.332100	-2.171800	-5.188200
O	-1.250000	1.158800	-1.969600
O	0.076900	2.021400	-4.030200
O	1.601500	0.755800	-1.897900
O	1.676200	-0.560600	-4.137100
O	-0.172100	-1.505000	-2.104900
O	-1.354100	-0.644300	-4.251900
H	-3.664500	-4.468800	-0.964800
H	-2.470400	-5.297500	-0.196300
H	-4.106900	-5.516600	0.240800
H	5.792200	-0.891700	-0.423500
H	5.812300	0.501400	0.450600
H	6.775300	-0.833400	0.908300
N	5.871300	-0.546000	0.538500
N	-3.404200	-4.836800	-0.043400
H	-3.379000	4.760300	0.426300
H	-2.762300	6.216100	1.076500
H	-2.073200	5.499700	-0.249400
N	-2.517300	5.321200	0.657100
Fe	0.100800	0.212000	-3.175300

Table S9. Optimized Cartesian coordinates (Å) of Δ-Fe(III)-[(DHB^{D}Lys^{D}Ser)$_3$].

N	-2.494900	-2.457300	-0.621000
N	-2.547800	0.674600	2.491100
H	-2.203000	-0.011500	1.835100
H	-2.162000	-1.536900	-0.870600
C	-1.866300	1.866900	2.477400
O	-2.336200	2.879600	2.953400
C	-0.563200	1.840400	1.671900
H	-0.850400	1.401300	0.707300
N	0.465800	0.959400	2.176900
H	1.265700	0.846400	1.527600
C	-1.821000	-3.035900	0.425800
O	-2.284600	-3.964000	1.055400
C	-0.525900	-2.317300	0.809200
H	-0.822600	-1.267200	0.948500
N	0.478200	-2.270700	-0.230800
H	1.268900	-1.643000	-0.007700
C	-1.827700	1.155200	-2.908700
O	-2.323400	1.085200	-4.014200
C	-0.528200	0.451000	-2.516000
H	-0.803400	-0.185900	-1.665300
N	0.498300	1.321900	-1.992700
H	1.287000	0.829900	-1.535000
C	0.045500	-2.783300	2.144600
C	-0.809200	-2.291500	3.299600
H	1.048900	-2.350800	2.232000
H	0.139700	-3.873200	2.155600
C	-0.145000	-2.458300	4.654700
H	-0.982500	-1.220300	3.135000
H	-1.780900	-2.796800	3.283400
C	-1.028300	-1.995800	5.797900
H	0.097400	-3.509900	4.838000
H	0.798300	-1.903700	4.665300
H	-1.905200	-2.635800	5.907800
H	-0.479600	-1.979500	6.739700
C	-0.020400	-0.500700	-3.593600
C	-0.899600	-1.741600	-3.653500
H	0.997000	-0.796200	-3.314100
H	0.022400	0.008100	-4.561300
C	-0.292400	-2.870400	-4.469300
H	-1.021700	-2.094000	-2.622200
H	-1.892900	-1.479900	-4.035800
C	-1.187900	-4.093300	-4.531700
H	-0.111900	-2.545400	-5.499000
H	0.678500	-3.143100	-4.041100
H	-2.106500	-3.890300	-5.085300
H	-0.675800	-4.937800	-4.993500
C	-0.031300	3.237800	1.366000
C	-0.880600	3.916100	0.301600
H	0.990100	3.120500	0.986400
H	0.008200	3.835500	2.281700
C	-0.268600	5.204100	-0.223000
H	-0.962200	3.215100	-0.535400
H	-1.891000	4.095700	0.688100
C	-1.158700	5.904500	-1.232800
H	-0.093600	5.908400	0.596700
H	0.704400	4.982500	-0.673900
H	-2.072300	6.276800	-0.765600
H	-0.640400	6.736200	-1.711500
C	0.868400	-3.330200	-0.979200
O	0.110600	-4.303800	-1.170400
C	0.843000	0.844500	3.469800
O	0.066900	1.134600	4.403700
C	0.860100	2.518100	-2.507200
O	0.096000	3.165300	-3.255700
C	2.180000	3.016200	-2.090300
C	3.115700	2.162300	-1.470800
C	2.527300	4.365100	-2.318400
C	4.369000	2.676000	-1.002100
C	3.747600	4.851200	-1.893800
H	1.816100	5.008500	-2.825500
C	4.666000	4.015000	-1.229000
H	4.004300	5.891900	-2.065500
H	5.616600	4.411000	-0.881800
C	2.195300	0.305700	3.684700
C	3.132300	0.254600	2.629500
C	2.572900	-0.164600	4.960700
C	4.412700	-0.359300	2.825300
C	3.821900	-0.718800	5.156700
H	1.862300	-0.088200	5.777000
C	4.738900	-0.827900	4.092600
H	4.101900	-1.086500	6.139100
H	5.710600	-1.288200	4.251000
C	2.209100	-3.226500	-1.572700
C	3.145500	-2.288400	-1.086700
C	2.579600	-4.080800	-2.634000
C	4.418000	-2.134300	-1.728500
C	3.820200	-3.955400	-3.226200
H	1.870800	-4.827700	-2.975800
C	4.736100	-2.979000	-2.785600
H	4.094900	-4.608500	-4.048900
H	5.701100	-2.872300	-3.274200
O	2.922700	0.885300	-1.275700
O	5.144300	1.813200	-0.372200
O	2.916100	0.730600	1.430900
O	5.181800	-0.440100	1.755200
O	2.935200	-1.507700	-0.060500
O	5.188800	-1.172300	-1.255700
H	-2.197700	4.240000	-1.965900
H	-2.093000	5.471700	-3.053000
H	-0.785500	4.441000	-2.758600
H	-2.226400	-3.859500	-2.728100
H	-2.079200	-5.430200	-3.193600
H	-0.784800	-4.597300	-2.494900
H	-2.205600	-0.579500	4.802900
H	-2.009700	-0.251300	6.401900
H	-0.785500	0.070800	5.284300
N	-1.536900	-0.608900	5.573400
N	-1.584800	4.975500	-2.322400
N	-1.596400	-4.533500	-3.164600
N	-2.472300	1.761900	-1.858500
H	-2.116900	1.508800	-0.947300
C	-4.650700	0.574200	-2.121400
C	-4.412400	2.547300	-0.631000
O	-5.788400	0.515500	-2.499900
O	-3.899700	-0.473400	-1.778600
H	-5.502600	2.565900	-0.608200
H	-4.027300	3.564100	-0.531800
O	-3.895400	1.774700	0.461600
C	-4.449200	-1.793500	-1.887500
C	-4.663200	1.598400	1.538800
H	-5.539500	-1.760000	-1.889400
H	-4.094600	-2.222800	-2.827800
C	-3.933700	-2.591200	-0.699300
O	-5.774200	2.034300	1.671700
C	-3.991900	0.708900	2.586600
C	-4.679100	-2.139600	0.560500
H	-4.211400	-3.637300	-0.846400
H	-4.300800	1.110300	3.554200
O	-5.790400	-2.504700	0.828700
O	-3.973800	-1.257600	1.268000
C	-4.552700	-0.697600	2.456900
H	-5.640900	-0.675900	2.383100
H	-4.258400	-1.315700	3.306400
C	-3.910800	1.897100	-1.910200
H	-4.199600	2.534400	-2.748700
Fe	4.257500	0.054900	0.038700

Table S10. Optimized Cartesian coordinates (Å) of Δ-Fe(III)-[(DHB^{L}Lys^{D}Ser)$_3$].

N	-1.226600	2.549900	2.237700
N	-1.607900	-2.331700	2.254200
H	-1.443100	-1.560700	1.621800
H	-0.649900	2.009200	1.607900
C	-1.234500	-3.571400	1.846100
O	-1.167100	-4.522200	2.610500
C	-0.807100	-3.694100	0.396800
H	-0.937500	-4.747800	0.129200
N	-1.522100	-2.835700	-0.510500
H	-0.981700	-2.216200	-1.139500
C	-2.487400	2.848200	1.832400
O	-3.340400	3.273000	2.596800
C	-2.811100	2.535000	0.384700
H	-3.656300	3.177200	0.116400
N	-1.709600	2.719900	-0.523000
H	-1.438300	1.940600	-1.147500
C	3.713600	0.727600	1.843200
O	4.499300	1.262600	2.610700
C	3.608700	1.162200	0.394300
H	4.587500	1.575000	0.129200
N	3.224900	0.115100	-0.515700
H	2.414400	0.266300	-1.141000
C	-3.191800	1.058500	0.260400
C	-4.353700	0.554200	1.097800
H	-2.296800	0.467600	0.496200
H	-3.409200	0.872700	-0.798200
C	-4.545400	-0.908300	0.730100
H	-4.138600	0.675800	2.164300
H	-5.262300	1.126600	0.880900
C	-5.628400	-1.645100	1.485100
H	-4.758800	-0.967300	-0.343600
H	-3.603000	-1.442300	0.887400
H	-5.329400	-1.884200	2.506900
H	-6.568600	-1.092600	1.515800
C	2.518200	2.227800	0.265800
C	2.663000	3.490000	1.096900
H	1.559800	1.749300	0.506300
H	2.464300	2.504200	-0.794000
C	1.493500	4.387700	0.726000
H	2.661700	3.248000	2.164700
H	3.613700	3.988500	0.877800
C	1.395900	5.692000	1.483800
H	1.549700	4.603500	-0.347300
H	0.559200	3.839000	0.881400
H	1.067300	5.545200	2.513900
H	2.337000	6.244100	1.492400
C	0.660000	-3.279300	0.268200
C	1.683200	-4.031000	1.100200
H	0.720600	-2.208900	0.505400
H	0.926700	-3.372700	-0.791400
C	3.045100	-3.480600	0.708600
H	1.483300	-3.895000	2.167800
H	1.631100	-5.105800	0.894700
C	4.227900	-4.040000	1.466300
H	3.192400	-3.658800	-0.363400
H	3.043700	-2.394500	0.841900
H	4.293700	-3.641800	2.480000
H	4.216500	-5.129900	1.517500
C	-1.228400	3.914900	-0.904600
O	-1.519600	4.957800	-0.274900
C	-2.794200	-3.024600	-0.899900
O	-3.553900	-3.797800	-0.272500
C	4.025700	-0.893400	-0.899500
O	5.073200	-1.162400	-0.267600
C	3.581400	-1.628800	-2.094000
C	2.419100	-1.254900	-2.805600
C	4.369100	-2.695700	-2.579100
C	2.070400	-1.926500	-4.026600
C	4.008800	-3.365900	-3.729400
H	5.278400	-2.955300	-2.047700
C	2.864100	-2.983600	-4.456500
H	4.620900	-4.185600	-4.093000
H	2.598000	-3.503000	-5.373300
C	-3.201600	-2.270300	-2.096100
C	-2.286300	-1.464000	-2.810000
C	-4.521900	-2.401700	-2.579600
C	-2.685900	-0.823400	-4.031800
C	-4.914200	-1.751400	-3.730900
H	-5.210300	-3.046200	-2.043400
C	-4.000300	-0.965500	-4.460400
H	-5.932200	-1.857600	-4.092900
H	-4.310900	-0.472500	-5.377800
C	-0.363700	3.894000	-2.095100
C	-0.113300	2.699400	-2.807200
C	0.175800	5.106600	-2.577800
C	0.646600	2.729400	-4.025900
C	0.937800	5.125800	-3.727200
H	-0.047000	6.024300	-2.043900
C	1.172900	3.942300	-4.454400
H	1.348900	6.063300	-4.089000
H	1.758100	3.968100	-5.369800
O	1.608900	-0.289200	-2.448600
O	1.002400	-1.471100	-4.653200
O	-1.041700	-1.260100	-2.454600
O	-1.749300	-0.139300	-4.660300
O	-0.555500	1.517600	-2.454000
O	0.781500	1.576900	-4.653200
H	6.299200	-3.702900	1.340900
H	5.623600	-4.230300	-0.060700
H	-0.015100	7.250300	1.436600
H	0.784300	7.038900	0.019000
H	-6.329300	-3.625800	1.391000
H	-6.500100	-2.792900	-0.014700
N	-5.882400	-2.941500	0.782700
N	5.473300	-3.628800	0.748700
N	0.369000	6.546000	0.808600
N	2.830000	-0.220900	2.247100
H	2.077600	-0.456200	1.614900
C	1.869000	0.702400	4.341100
C	2.014600	-1.762000	3.984900
O	1.837800	0.829500	5.535500
O	1.241400	1.493200	3.462200
H	2.010300	-1.912100	5.064500
H	2.575400	-2.558200	3.494300
O	0.675800	-1.825900	3.471500
C	0.519600	2.625900	3.969200
C	-0.328000	-1.950200	4.348100
H	0.655700	2.705800	5.047700
H	0.926300	3.505400	3.469300
C	-0.954000	2.495600	3.646600
O	-0.208000	-1.959800	5.543700
C	-1.686700	-2.068000	3.663300
C	-1.536200	1.269000	4.342600
H	-1.448900	3.349900	4.117700
H	-2.169900	-2.926100	4.139400
O	-1.608800	1.180300	5.538800
O	-1.927700	0.330500	3.472600
C	-2.538300	-0.859800	3.992800
H	-2.660700	-0.778300	5.072800
H	-3.510200	-0.951100	3.507000
C	2.640700	-0.422500	3.656500
H	3.626000	-0.419100	4.131700
Fe	0.007600	-0.011100	-3.699400
H	5.386900	-2.637800	0.379700
H	-0.420900	5.955500	0.416900
H	-4.980200	-3.345900	0.397800

Table S11. Optimized Cartesian coordinates (Å) of Δ-Fe(III)-[(DHB^{D}Lys^{L}Ser)$_3$].

O	2.708200	-0.681000	5.512500
O	-0.777000	2.348600	5.647900
O	-1.859900	-1.974000	5.567700
C	2.231900	-0.365600	4.454600
C	-0.803700	2.001500	4.497100
C	-1.444100	-1.767400	4.458800
C	1.564700	2.028000	4.314200
C	-2.588800	0.306600	4.293600
C	0.949800	-2.352400	4.330800
H	1.506100	1.990500	5.402900
H	1.904800	3.013500	3.993000
H	-2.551400	0.263600	5.382800
H	-3.606700	0.126200	3.946800
H	0.933400	-2.298300	5.420500
H	1.663000	-3.111700	4.006300
O	-1.731800	-0.696000	3.719900
O	1.343700	-1.087200	3.772500
O	0.269100	1.799300	3.731700
C	-0.439600	-2.681900	3.783900
H	-0.665200	-3.698800	4.110700
C	-2.105100	1.667300	3.790900
H	-2.842600	2.396700	4.132600
C	2.526500	0.962800	3.782600
H	3.524000	1.262800	4.108400
N	-2.014800	1.712500	2.341100
N	2.498800	0.881500	2.330900
N	-0.482200	-2.620700	2.332700
H	-0.571800	-1.691000	1.948600
H	-1.164600	1.330800	1.952700
H	1.734000	0.341700	1.951900
C	0.341000	-3.473400	1.639200
O	0.726600	-4.520700	2.120900
C	0.847000	-2.929900	0.301900
H	1.230600	-1.931800	0.532100
N	-0.178300	-2.681000	-0.682300
H	0.057900	-1.997900	-1.420800
C	-3.158200	1.458000	1.624200
O	-4.262700	1.662100	2.089100
C	-2.928300	0.763500	0.283000
H	-2.265900	-0.076400	0.514100
N	-2.180600	1.533500	-0.681700
H	-1.710700	0.982800	-1.419400
C	2.832300	2.012200	1.624500
O	3.553100	2.869500	2.095800
C	2.108400	2.165000	0.288000
H	1.052800	2.002200	0.526600
N	2.403300	1.141800	-0.686200
H	1.691900	1.009400	-1.423900
C	-4.215600	0.190900	-0.301600
C	-4.775300	-0.983300	0.500500
H	-3.969200	-0.156100	-1.311400
H	-4.958600	0.988400	-0.389600
C	-3.722700	-2.059100	0.755600
H	-5.181700	-0.631800	1.453600
H	-5.606500	-1.417900	-0.066100
C	-4.344100	-3.360600	1.210000
H	-3.148500	-2.231400	-0.162200
H	-3.018300	-1.726500	1.528000
H	-4.977800	-3.218200	2.087400
H	-4.935800	-3.818100	0.415200
C	2.246000	3.572100	-0.285100
C	1.507200	4.632400	0.530000
H	1.819400	3.539900	-1.293800
H	3.306600	3.822800	-0.374500
C	0.048400	4.256800	0.770700
H	2.009600	4.791500	1.488600
H	1.550500	5.578800	-0.021000
C	-0.769900	5.434100	1.251300
H	-0.385000	3.866200	-0.157300
H	-0.021500	3.464500	1.525400
H	-0.332200	5.891900	2.140400
H	-0.872600	6.193200	0.473900
C	2.005500	-3.755900	-0.251000
C	3.291000	-3.608000	0.561700
H	2.186500	-3.402600	-1.272200
H	1.704700	-4.805800	-0.304700
C	3.737700	-2.155000	0.679700
H	3.156400	-4.034800	1.560500
H	4.078800	-4.187200	0.066700
C	5.120300	-2.031100	1.282000
H	3.726100	-1.685400	-0.310200
H	3.046900	-1.592500	1.317200
H	5.205500	-2.593300	2.214100
H	5.893300	-2.370700	0.590700
C	-2.470300	2.788300	-1.069900
O	-3.179400	3.544800	-0.366900
C	-1.145700	-3.539300	-1.044900
O	-1.452100	-4.521300	-0.328800
C	3.635900	0.765900	-1.072300
O	4.644000	1.007500	-0.370000
C	3.705800	0.021100	-2.338700
C	2.609300	-0.019600	-3.226800
C	4.897200	-0.656700	-2.679600
C	2.700600	-0.755400	-4.456700
C	4.981300	-1.362000	-3.862600
H	5.736000	-0.609100	-1.993100
C	3.888000	-1.414300	-4.751200
H	5.897000	-1.887600	-4.116000
H	3.961600	-1.977300	-5.678100
C	-1.839400	-3.222600	-2.302600
C	-1.330900	-2.256900	-3.198700
C	-3.028200	-3.911200	-2.630500
C	-2.019900	-1.974300	-4.426700
C	-3.690000	-3.633200	-3.808800
H	-3.403200	-4.658900	-1.939400
C	-3.189900	-2.669200	-4.707800
H	-4.607500	-4.160600	-4.052200
H	-3.719200	-2.456200	-5.633100
C	-1.863000	3.214900	-2.338900
C	-1.291800	2.277900	-3.227700
C	-1.869000	4.583600	-2.687200
C	-0.713700	2.714800	-4.467600
C	-1.310400	4.999600	-3.878100
H	-2.319300	5.292100	-1.999800
C	-0.734400	4.071000	-4.769300
H	-1.309700	6.054100	-4.137000
H	-0.293200	4.409600	-5.703200
O	1.467700	0.582700	-3.014300
O	1.618400	-0.754400	-5.213600
O	-0.236700	-1.569500	-2.996700
O	-1.476500	-1.048100	-5.195700
O	-1.241800	0.989600	-3.006700
O	-0.187300	1.769500	-5.225100
H	4.762300	-0.262500	2.311800
H	6.353700	-0.461800	1.918900
H	5.218700	0.015900	0.749200
H	-2.102900	4.286900	2.355000
H	-2.734100	5.745200	1.908300
H	-2.597300	4.482100	0.783100
H	-2.710200	-3.964900	2.342800
H	-3.659100	-5.226100	1.864000
N	-3.273600	-4.328500	1.574600
N	5.399500	-0.600900	1.590400
N	-2.141400	4.974900	1.603300
Fe	-0.009400	-0.006800	-4.307200
H	-2.601100	-4.477400	0.767500
